# Generative models improve fairness of medical classifiers under distribution shifts

**DOI:** 10.1038/s41591-024-02838-6

**Published:** 2024-04-10

**Authors:** Ira Ktena, Olivia Wiles, Isabela Albuquerque, Sylvestre-Alvise Rebuffi, Ryutaro Tanno, Abhijit Guha Roy, Shekoofeh Azizi, Danielle Belgrave, Pushmeet Kohli, Taylan Cemgil, Alan Karthikesalingam, Sven Gowal

**Affiliations:** 1 Google DeepMind, London, UK; 2 Google Research, London, UK; 3 GSK.ai, London, UK

**Keywords:** Medical imaging, Diagnosis

## Abstract

Domain generalization is a ubiquitous challenge for machine learning in healthcare. Model performance in real-world conditions might be lower than expected because of discrepancies between the data encountered during deployment and development. Underrepresentation of some groups or conditions during model development is a common cause of this phenomenon. This challenge is often not readily addressed by targeted data acquisition and ‘labeling’ by expert clinicians, which can be prohibitively expensive or practically impossible because of the rarity of conditions or the available clinical expertise. We hypothesize that advances in generative artificial intelligence can help mitigate this unmet need in a steerable fashion, enriching our training dataset with synthetic examples that address shortfalls of underrepresented conditions or subgroups. We show that diffusion models can automatically learn realistic augmentations from data in a label-efficient manner. We demonstrate that learned augmentations make models more robust and statistically fair in-distribution and out of distribution. To evaluate the generality of our approach, we studied three distinct medical imaging contexts of varying difficulty: (1) histopathology, (2) chest X-ray and (3) dermatology images. Complementing real samples with synthetic ones improved the robustness of models in all three medical tasks and increased fairness by improving the accuracy of clinical diagnosis within underrepresented groups, especially out of distribution.

## Abstract (web summary)

By generating synthetic image samples specific to underrepresented groups, diffusion models help medical image classifiers to achieve greater fairness metrics across a variety of medical disciplines and demographic attributes.

## Main

The advent of machine learning (ML) in healthcare promises advances in care in a wide range of applications^1–3^. Artificial intelligence (AI) dermatological tools (for example, refs. ^1,4^) have the potential to allow patients to assess their conditions better and improve diagnostic accuracy^5^. Similarly, ML technologies have unlocked new capabilities in computational pathology that have the ability to handle the gigantic quantity of data created throughout the patient care lifecycle and improve classification, prediction and prognostication of diseases^5,6^. These solutions are often motivated by the global shortage of expert clinicians, for example, in the case of radiologists^7^, and demonstrate that ML models can facilitate the detection of conditions^8^. Despite these rapid methodological developments and the promise of transformative impact^9^, few of these approaches (if any) have yet achieved widespread adoption and scaled impact on clinical outcomes^10^. One major barrier to adoption is the brittle degradation in performance of medical ML systems caused by ‘out-of-distribution’ data: discrepancies between the populations, diseases, acquisition technologies or environments used to train medical ML systems and those encountered during deployment. As ref. ^11^ highlighted, only 24% of published studies evaluate the performance of their proposed algorithms on external cohorts or compare this out-of-distribution performance with that of clinical experts. Many studies do not validate the efficacy of algorithms in multiple settings; the ones that do often perform poorly when introduced to new environments not represented in the training data.

In addition to this challenge of out-of-distribution generalization, underrepresentation of specific groups, conditions or hospitals also causes notable challenges of fairness and equity even when systems are deployed in datasets mirroring their training environment, with lower performance typically seen in rarer groups, conditions, individuals or their intersections. Previous work showed that a developed model may perform unexpectedly poorly on underrepresented populations or population subgroups in radiology^12,13^, histopathology^14^ and dermatology^15^. However, the issues of robustness to distribution shifts and statistical fairness have rarely been tackled together. Building a method that is robust across populations and subgroups, such that model performance does not degrade and benefits can be transferred when applied across groups, is a nontrivial task. This is because of data scarcity^16^, challenges in the acquisition strategies of evaluation datasets (for example, different imaging or screening protocols^10,17,18^) and the limitations of evaluation metrics^10^.

**Fig. 1:**
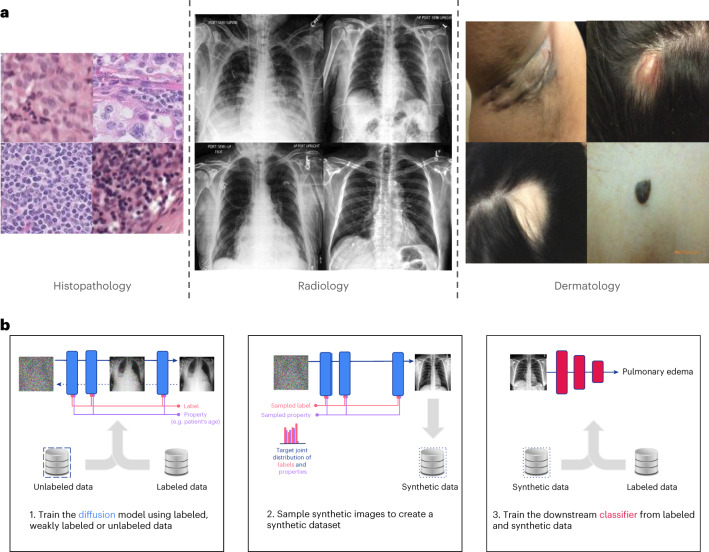
Generated samples and method overview. **a**, Samples generated by our conditional diffusion model for the different imaging modalities. **b**, Method overview. In the proposed approach, we first trained a diffusion model on both labeled and unlabeled data (if available). In a general setting, unlabeled data may consist of in-distribution or OOD data (for example, from an unseen hospital) for which expert labels are not available. Subsequently, we sampled synthetic images from the diffusion model according to particular specifications (for example, an image of a female individual with pulmonary edema). Finally, we trained a downstream diagnostic model on a combination of the real labeled images and the synthetic images sampled from the diffusion model. The dotted outlines represent synthetic data, while the dashed outlines represent unlabeled data.

In this work, we leveraged diffusion models^19,20^ and potentially available unlabeled data to capture the underlying data distribution and augment real samples when training diagnostic models across these three modalities. We showed that combining synthetic and real data can lead to significant improvements in diagnostic accuracy, while closing the fairness gap with respect to different attributes under distribution shifts. While we do not propose this approach as a replacement for high-quality and representative data collection strategies, we posit that, in the absence of additional resources, it allows practitioners to make the most of their available labeled and unlabeled data to close potentially harmful gaps in diagnostic accuracy between overrepresented and underrepresented populations without penalizing the former. Finally, we showed that diffusion models can generate high-quality images (Fig. 1a) across modalities and performed an in-depth analysis to shed light on the mechanisms that improve the generalization capabilities of the downstream classifiers (Methods, ‘In-depth analysis for dermatology’). This capability was further validated by an evaluation of synthetic images by expert dermatologists, yielding diagnostic accuracy comparable to when diagnosing real images.

## Results

### Overview of the proposed approach and experimental setting

Our proposed approach, illustrated in Fig. 1b, leverages diffusion models for learning augmentations of the data to improve the robustness and fairness of medical ML models. We viewed learned augmentations as a means of enriching our training dataset with the goal of making it more diverse in a steerable and configurable way. Our approach consisted of three main steps: (1) we train a generative model given the available labeled and unlabeled data; we assumed that labeled data were available only for a single source domain (for example, a particular hospital with a specific scanner or imaging protocol), while additional unlabeled data could be from any domain (in-distribution or out of distribution (OOD), for example, data from multiple hospitals, a subset of which was not labeled by experts because of limited resources). We either conditioned the generative model only on the diagnostic label or on both the diagnostic label and a property (for example, hospital ID or sensitive attribute label). We borrowed the term ‘sensitive attribute’ from the fairness literature to describe demographic attributes (for example, sex, ethnicity or age) we wanted the model to be fair against. All of the data used in this research were de-identified before authors gained access to it. Conditioning the model on either or both of these attributes allowed us to configure the synthetic examples that we wanted to use to enrich our training set. If high-resolution images are required (more than 96 × 96 resolution), we further trained an upsampling diffusion model in a similar manner. It is worth highlighting that both the low-resolution generative model and the upsampler were trained with the same conditioning vector (that is, either with label or label and property conditioning); (2) we sampled from the generative model according to a sampling strategy. In our experiments, we assumed that uniform representation of different values of an attribute constitutes a fair strategy, for example, for each condition it is equally likely to observe an image of a male and a female individual, or from a particular hospital. To do this, we sampled uniformly from the attribute distribution and preserved the original diagnostic label distribution to preserve the original disease prevalence. Sampling multiple times from the generative model allowed us to obtain different augmentations for a given condition (and property), consequently increasing the diversity of training samples for the downstream classifier; (3) we enriched our original training dataset from the source domain with the synthetic images sampled from the generative model and trained a diagnostic model (potentially for multiple labels, if more than one condition is present at once). We provide the exact details of the experimental setting for each modality in the Methods (‘Experimental setting for each modality’).

#### Experimental protocol

We evaluated this approach using denoising diffusion probabilistic models (DDPMs) on different medical contexts and tracked diagnostic performance (for example, top-1 accuracy) and fairness in-distribution and OOD. We considered in-distribution datasets as consisting of images from the same demographic and disease distribution and acquired with the same imaging protocol as the training set. Out-of-distribution datasets may differ from the training set in any or all of those dimensions. Evaluation of the out-of-distribution datasets is equivalent to developing an ML model on a certain population (for example, from a particular hospital or geographical location) and testing its performance on a population from an unseen hospital or acquired under new conditions. Across all settings, the diagnostic and diffusion models were trained with the same labeled data. We provide more details about this and a summary of the setting used for each modality in the Methods (‘Overview of methodology’).

#### Evaluation metrics

To measure the performance of the different baselines and the proposed method, we used two sets of metrics: one set was more focused on diagnostic accuracy (that is, top-1 accuracy for histopathology, receiver operating characteristic (ROC)-area under the curve (AUC) for radiology and high-risk sensitivity for dermatology), while the second set was more geared toward fairness (see summary in Table 1). The performance metrics varied depending on the classification task performed for each modality (that is, binary versus multiclass versus multilabel) and considered label imbalance. High-risk sensitivity captured the true positive rate for the high-risk conditions and was deemed the most relevant for the diagnostic tool by expert dermatologists. For fairness, we looked at the performance gap (depending on the metric of interest) in the binary attribute setting and the difference between the worst and best subgroup performance for categorical attributes, for example, hospital ID and ethnicity. For continuous sensitive attributes, like age, we discretized them into appropriate buckets (Methods and Extended Data Table 1).

### Clinical tasks and datasets

#### Histopathology

The first setting we considered is histopathology. Variation in staining procedures in different hospitals leads to distribution shifts that can challenge an ML model that has only encountered images from a particular hospital. The cancer metastases in lymph nodes challenge (CAMELYON17) by Bandi et al.^21^ aims to improve generalization capabilities of automated solutions and reduce the workload on pathologists who have to manually label those cases. The corresponding dataset contains images from five different hospitals and the task was to predict whether the histological lymph node sections captured by the images contain cancerous cells, indicating breast cancer metastases (as posed by the WILDS challenge^22^). Two of the hospital datasets provided by the challenge were held out for out-of-distribution evaluation and three were considered in-distribution datasets because of similar staining procedures. We considered this as the simplest setting for our experiments because there was no extreme disease prevalence or demographic shifts. The labeled dataset contained 455,954 patches, while the unlabeled dataset contained 1.8 million patches from the three training hospitals; full statistics are given in Methods and Extended Data Table 1a. The unlabeled dataset contained the hospital identifier but not the diagnostic label.

To understand the impact of the number of labeled examples on fairness and overall performance, we created different variants of the labeled training set, where we varied the number of samples from two of the three training hospitals (3 and 4). The number of labeled examples from one hospital remained constant. We compared top-level classification accuracy and fairness gap, that is, the accuracy gap between the best and worst performing hospital across the in-distribution hospitals, to different baselines (more details about the baselines are provided in Methods (‘Baselines’)).

**Fig. 2:**
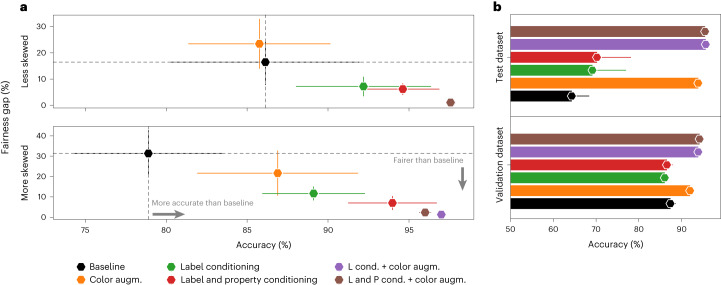
Results on histopathology dataset. **a**, In-distribution fairness gap (in percentage) between the best and worst performing hospital versus overall prediction accuracy for the presence of breast cancer metastases in histopathology images on *n* = 33,560 samples. In the less skewed setting, we included 1,000 labeled samples from hospitals 3 and 4, while in the more skewed setting we included only 100 samples. **b**, OOD distribution results on *n* = 85,054 samples. Prediction accuracy (*x* axis) on the validation and test hospitals when training the generative model on all in-distribution labeled examples is shown. Note that the validation set was used for model selection, given that its distribution was more similar to the training distribution. We compared the following methods: baseline model with no augmentations; ‘Color augm.’ for a model that uses color augmentations; ‘Label conditioning’ and ‘Label and property conditioning’ for our proposed approach of a generative model conditioned on the diagnostic label and both the diagnostic label and the hospital ID, respectively; ‘L cond. + color augm.’ and ‘L and P cond. + color augm.’ were used to apply color augmentations on the images generated with the diffusion models. Combining color augmentation with synthetic data performed best across all settings. Data are presented as mean ± s.d. across five technical replicates.

We found that using synthetic data outperformed both in-distribution baselines in the less skewed (with 1,000 labeled samples from hospitals 3 and 4) and more skewed setting (with only 100 labeled samples) while closing the fairness gap between hospitals. We obtained the best accuracy OOD when using all in-distribution labeled examples as shown in Fig. 2b (in the OOD setting, there were one validation and one test hospital, so we do not report a performance gap). We found that performing color augmentation on top of the generated samples generalized best overall, leading to a 48.5% relative improvement over the baseline model and 3.2% over the model trained with color augmentations on the test hospital, while reducing the performance gap between in-domain hospitals by 20 absolute percentage points.

**Extended Data Fig. 1:**
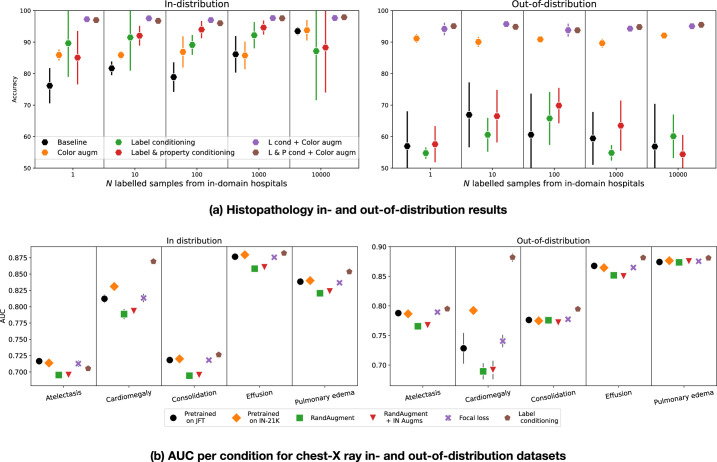
Additional results for histopathology and radiology. (a) Prediction accuracy for the presence of breast cancer metastases in whole-slide images of histological lymph node sections. In these experiments we vary the number of labeled samples N used to train the diffusion model from in-distribution datasets on in-distribution and out-of-distribution hospitals. Performance improvements are particularly striking in the low data regime (that is, small values of N). Combining color augmentation with synthetic data performs best across all settings (in and out-of-distribution). (b) Additional results for chest radiology. Data are presented as mean values ± SD across 5 technical replicates.

This validated that we can indeed use synthetic data to better model the data distribution and outperform variants using real data alone. We also observed that this method was most effective in a low-data regime (that is, the more skewed setting in Fig. 2a), while being able to recover performance that other approaches achieve with 100× more labeled samples, as shown in Extended Data Fig. 1a. This translates to more significant improvements in scenarios where we only have access to a few labeled examples from a particular hospital or population because of limited resources.

#### Chest radiology

The second setting we considered is radiology. We focused our analysis on two large public radiology datasets, CheXpert^23^ and ChestX-ray14 (National Institutes of Health)^24^. These datasets have been widely studied^8,12,13^ for model development and fairness analyses. For these datasets, demographic attributes like sex and age are publicly available; classification was performed at a higher resolution, that is, 224 × 224 as in ref. ^25^. After training the generative and diagnostic models on 201,055 examples of chest X-rays from the CheXpert dataset, we evaluated on a held-out CheXpert test set (containing 13,332 images), which we considered in-distribution, and the test set of ChestX-ray14 (containing 17,723 images), which we considered OOD because of demographic and acquisition shifts. We focused on five conditions for which labels existed in common between the two datasets, that is, atelectasis, consolidation, cardiomegaly, pleural effusion and pulmonary edema, while each of these datasets contained more conditions (not necessarily overlapping), as well as examples with no findings, corresponding to healthy controls. Note that the labeling procedures for the two datasets were defined and enacted separately, which probably increased the complexity of the task. In this setting, the model backbone was shared across all conditions, while a separate (binary classification) head was trained for each condition, given that multiple conditions can be present at once. We report the ROC-AUC curve in line with the CheXpert leaderboard.

**Fig. 3:**
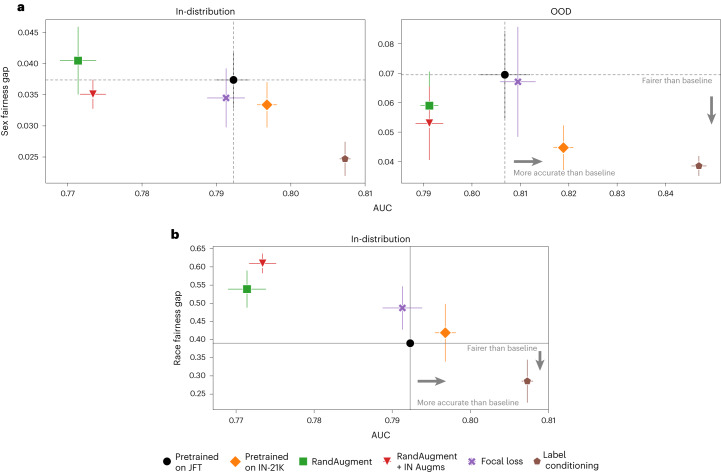
Results on chest radiology datasets. **a**,**b**, Comparison of average AUC versus fairness (AUC) gap across different baselines for radiology for sex (**a**) and race (**b**) for in-distribution (*n* = 23,261 samples) and OOD (*n* = 17,723 samples) datasets. Race labels are not available for the OOD dataset. For **a**, we report results in-distribution (left) and OOD (right) on CheXpert and ChestX-ray14 datasets, respectively. We marked the baseline ‘Pretrained on JFT’ with black. Label conditioning corresponds to the model that used synthetic images from a diffusion model conditioned on only the diagnostic labels. We further compared to other strong contenders, that is, a BiT-ResNet model pretrained on ImageNet-21K (Pretrained on IN-21K), a model pretrained on JFT using RandAugment heuristic augmentations (RandAugment), a model trained with RandAugment on top of standard ImageNet augmentations (RandAugment + IN Augms) and a model trained with focal loss (Focal loss). To ensure a fair comparison, all methods were trained and finetuned for the same number of steps and with the same batch size. For the fairness gap, smaller values are preferable. Data are presented as the mean ± s.d. across five technical replicates.

**Extended Data Fig. 2:**
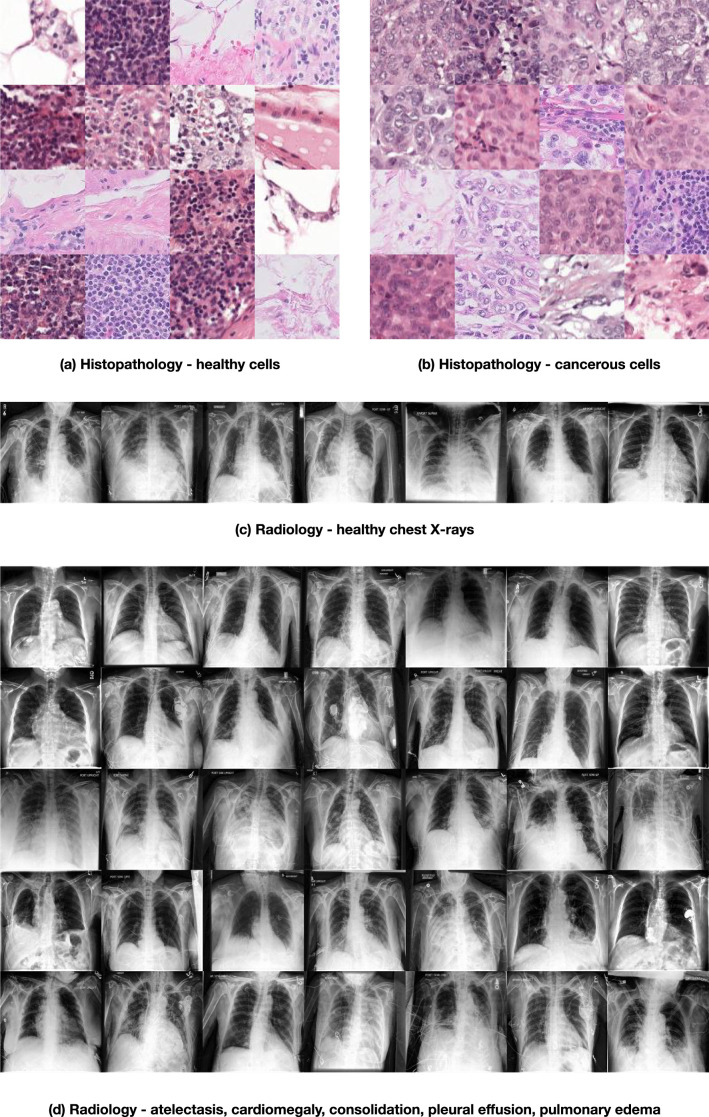
Generated images in simple settings. (a) Synthetic images for healthy cells in histopathology. (b) Synthetic images for cancerous cells in histopathology. (c) Synthetic images of chest X-rays for healthy individuals. (d) Synthetic images of chest X-rays with the following conditions: atelectasis, cardiomegaly, consolidation, pleural effusion, pulmonary edema (one condition per row).

We observed that synthetic images improved the average AUC for the five conditions of interest in-distribution, but even more so OOD (Fig. 3a). Improvements were particularly striking for cardiomegaly, where the model trained purely with synthetic images improved the AUC by 21.1% (Fig. 3a). Overall, we observed a relative improvement of 5.2% on average AUC OOD and a 44.6% improvement in sex fairness gap. We also observed a 31.7% decrease in race fairness gap in-distribution (Fig. 3b). We show some examples of synthetic images for a model conditioned on the diagnostic label in Extended Data Fig. 2c,d.

#### Dermatology

For the dermatology setting, we considered a dermatology dataset of images grouped into 27 labeled conditions ranging from low risk (for example, acne, verruca vulgaris) to high risk (for example, melanoma). Out of these conditions, three were considered to be high risk: basal cell carcinoma; melanoma; and squamous cell carcinoma (SCC) and squamous cell carcinoma in situ (SCCIS). For the purposes of our experiments, we considered three datasets: the in-distribution dataset featuring 16,530 cases from a teledermatology dataset acquired from a population in the United States (Hawaii and California); the OOD 1 dataset featuring 6,639 images of clinical type focusing mostly on high-risk conditions from an Australian population; and OOD 2 featuring 3,900 teledermatology images acquired in Colombia. To train the downstream classifier, we used labeled samples from only one of these datasets (in-distribution), while we included unlabeled images from the other two distributions when training the diffusion model. We evaluated on a held-out slice of the in-distribution dataset and two OOD sets to investigate how well models generalized. We present results for the OOD 2 dataset in Supplementary Information, Additional results for dermatology, because it has similar label distribution to the in-distribution dataset and is less challenging.

We explored whether the proposed approach can be used to not only improve OOD accuracy but also fairness over the different label predictions and attributes for the in-distribution dataset. While the datasets were already imbalanced with respect to different labels and sensitive attributes, we also investigated how the performance varied as a dataset becomes more or less skewed along a single one of these axes. This allowed us to better understand to what extent conditioning generative models on the axis of interest can help alleviate biases with regard to the corresponding attribute.

**Fig. 4:**
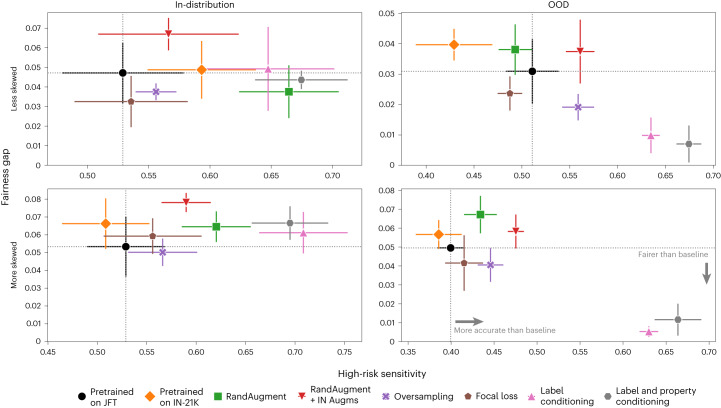
Results on dermatology datasets. Comparison of high-risk sensitivity (for basal cell carcinoma, melanoma and SCC/SCCIS) versus fairness gap with regard to sex in dermatology across different baselines. We report results in-distribution (left) and OOD (right) for OOD 1, as well as for the less skewed (top) and more skewed (bottom) setting. We marked the baseline ‘Pretrained on JFT’ with black. ‘Label conditioning’ and ‘Label and property conditioning’ correspond to the models that used synthetic images sampled from a diffusion model conditioned on only the label, and the label and sensitive attribute, respectively. We further compared to other strong contenders, that is, a BiT-ResNet model pretrained on ImageNet-21K (Pretrained on IN-21K), a model pretrained on JFT using RandAugment heuristic augmentations (RandAugment), a model trained with RandAugment on top of standard ImageNet augmentations (RandAugment + IN Augms), a model trained on a resampled version of the training dataset that is more balanced with regard to the sensitive attribute (Oversampling) and a model trained with focal loss (Focal loss). To ensure a fair comparison, all methods were trained and finetuned for the same number of steps and with the same batch size. For the fairness gap, smaller values are preferable. There are *n* = 1,349 samples in the in-distribution dataset and *n* = 6,639 samples in the OOD dataset. Data are presented as the mean ± s.d. across five technical replicates.

In Fig. 4, we illustrate how different methods compare for a single axis of interest with regard to sensitivity for the three high-risk conditions mentioned above and fairness. In the more skewed setting, the training dataset contained a maximum of 100 samples from the underrepresented subgroup regardless of the underlying condition, while in the less skewed setting it contained a maximum of 1,000 samples. We compared all methods in the four different settings: in-distribution and OOD, as well as less and more skewed with respect to the sensitive attribute of interest, that is, sex. We observed that in all settings, combining heuristic augmentations improved the predictive performance across the board, but harmed fairness of the model. Using RandAugment alone was beneficial for high-risk sensitivity in-distribution, but not OOD, but it harmed fairness in the OOD setting. Oversampling slightly closed the fairness gap across the board while improving performance, as expected. The approaches that leverage synthetic data, ‘Label conditioning’ and ‘Label and property conditioning’, improved on high-risk sensitivity in-distribution without reducing fairness, while they yielded a significant improvement in the OOD setting on both axes. In the more skewed setting, in particular, ‘Label and property conditioning’ led to 27.3% better high-risk sensitivity compared to the baseline in-distribution and a striking 63.5% OOD, while closing the fairness gap by 7.5× OOD. It is worth noting that the underrepresented group in the training set and the ID evaluation set was overrepresented in the OOD evaluation set. Our approach showed improvements in accuracy and fairness metrics with respect to different sensitive attributes, while being able to generalize these improvements OOD as shown in Methods, ‘Additional results’. The strong overall performance and reduced fairness gap OOD indicates that the diagnostic model learned better generalizable features when leveraging synthetic data.

## Discussion

In this work, we propose using conditional diffusion models to improve the robustness and fairness of ML systems applied to medical imaging. More specifically, we show that diffusion models can produce useful synthetic images in three different medical settings of varying difficulty, complexity and resolution: histopathology, radiology and dermatology. Our experimental evaluation provides extensive evidence that synthetic images can indeed improve statistical fairness, balanced accuracy and high-risk sensitivity in a multiclass setting, while improving the robustness of models both in-distribution and OOD. In fact, we observe that generated data can be more beneficial OOD than in-distribution even in the absence of data from the target domain during training of the generative model (in the case of radiology). Generative models were label-efficient in both histopathology and dermatology settings, where we demonstrate that only a few labeled examples are sufficient for the diffusion models to capture the underlying data distribution well. This is particularly impactful in the medical setting, where data for particular conditions or demographic subgroups can be scarce or, even when available, acquiring expert labels can be expensive and time-consuming. For the reader that is familiar with regularization techniques, we view diffusion models as another form of regularization, which can be combined with any other architecture or learning method improvements.

**Fig. 5:**
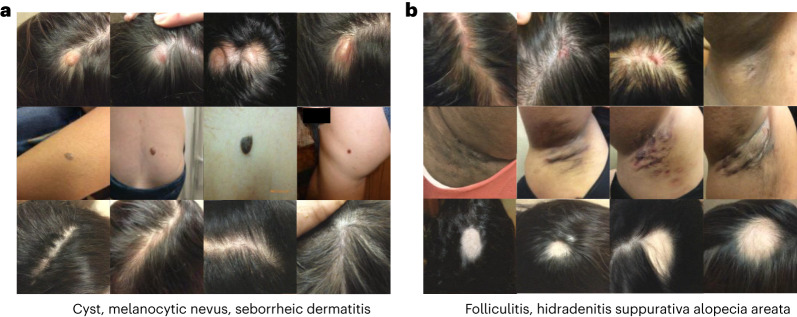
Generated images in the dermatology setting. Each row of images corresponds to a different condition. **a**, Generated images for cyst, melanocytic nevus and seborrheic dermatitis. **b**, Generated images for folliculitis, hidradenitis and alopecia areata.

Even though we did not make any assumptions when training the diffusion model, we found interesting dynamics when combining real and synthetic data. In certain settings, that is, histopathology and radiology, we observed that we can rely purely on generated data and still outperform baselines trained with real labeled data (Methods, ‘Additional results’). In other settings, like dermatology, we observed that real data were more essential for training of the downstream discriminative model. We took this a step further and analyzed the impact of generated data and the mechanisms underlying the improvements in robustness and fairness that we report. In-depth analysis in one of the modalities indicated that synthetic samples from a diffusion model yield diverse (Fig. 5), realistic and canonical images deemed diagnosable by expert clinicians to a great extent (Methods, ‘In-depth analysis for dermatology’). Synthetic samples seem to better align distributions of different domains, while at the same time allowing models to learn more complex decision boundaries that reduce their reliance on spurious correlations. Finally, we highlight some practical benefits and discuss a number of potential risks and limitations from relying on generated data.

First, synthetic data are reusable. Beyond the analysis and utility of synthetic data for the particular tasks that we considered in this work, there are many other potential applications for which they can be useful. The same synthetic data can be used for data augmentation across different models and, potentially, tasks. For example, handcrafted augmentations are often used to introduce invariances and learn better representations in a self-supervised manner for a variety of downstream tasks.

Furthermore, the proposed approach is scalable. As we demonstrate in the Supplementary Information, if we have a perfect generative model, then we can perform perfectly under the fair distribution. Moreover, the better the generative model, the more our results should improve. Thus, as generative modeling improves or as more data are available, results should improve accordingly.

Combining this technique with privacy-preserving technologies holds significant promise for the medical field. Principles of data governance, confidentiality, privacy and consent are vital in healthcare, but may be associated with relative limitations of data availability for the training of ML models in underrepresented groups. There is preliminary evidence that federated learning can be used to learn classification models from multiple institutions^26^; if it were possible to generate private synthetic data, these synthetic data could be used for data augmentation along with a smaller, public dataset to improve performance. This could have practical benefits when data sharing to protect personally identifiable information while achieving high-quality performance. Such an approach would of course be associated with its own risks, some of which are discussed in ref. ^27^.

Even though we showed that diffusion models can be particularly label-efficient, this should not encourage practitioners to abandon their data and label acquisition efforts; nor does it imply that generated data can replace real data under any circumstances. What this research demonstrates is that, when labeled data and resources are limited, there are ways to make more of the available labeled and unlabeled data. There is also the potential that using generative models may lead to overconfidence in an AI system because images look realistic to a nonexpert. Additional data collection will always be important, along with comprehensive analysis of the underlying data and their caveats. Synthetic data from a generative model should only be used as a complement to additional data collection and accompanied by rigorous evaluation on real data, ideally outside the main source domain to understand the generalization capabilities of the models. In other words, synthetic data are one solution to increase diversity, but not a substitution of efforts to increase data representation for underrepresented conditions and populations.

If the generative model is of poor quality or biased, then we may end up exacerbating problems of bias or structural inequities in the downstream model. The generative model may be unable to generate images of a certain label and sensitive attribute. In other settings, the model may always generate a specific part of the distribution for a certain label and sensitive attribute instead of capturing the true image distribution. The generative model may also create incorrect images of a given label and sensitive attribute, leading the classification model to make mistakes confidently in those regions. Medical training datasets can also encode structural inequities in the delivery of healthcare, which could be propagated by generative models in ways that might not be immediately apparent on inspection of the synthetic examples being created. Therefore, it is particularly important that evaluation data remain unbiased and that multiple safeguards are implemented to assess model fairness and mitigate the multifaceted nontechnical health inequities that cannot be addressed solely by data curation and model development.

Another important risk to be mindful of is that the insights that we obtain by analyzing the model are only as good as our evaluation setup. If the evaluation datasets are not diverse enough, do not capture high-risk conditions well or are not representative of the population, then any conclusions we draw from these results will be limited. Therefore, care needs to be taken to report and understand what each of the evaluation setups is capturing. For example, as Varoquaux et al.^10^ highlight, clinician-level performance is often overstated without validating models OOD. Moreover, clinical applicability of patch-level evaluation for the histopathology setting can be limited and whole-slide image analysis should be investigated further.

In terms of limitations, sensitive attributes are not always observed or explicitly tracked and reported^28^, often to protect people’s privacy. At the same time, the way labels are assigned may have its own limitations. For example, using binary gender and sex attributes (or using the two interchangeably) does not represent people that identify as nonbinary. Similarly, researchers have criticized the Fitzpatrick skin type because it is less accurate on shades of darker skin tones, which could cause models to misidentify or misrepresent people with darker skin. Similarly, there are other unobserved characteristics that can influence disease and are not accounted for in a visual image of skin, for example, social determinants of health. One instance of this is how dermatitis in a person who lives in a communal setting could have a different differential diagnosis than dermatitis in a high-income setting on a high-income individual. These are important considerations when relying on such attributes to condition learned augmentations or to perform fairness analyses.

Finally, synthetic images should be handled with caution and transparency because they may perpetuate biases in the original training data. It is important to tag and identify when a synthetic image has been added to a database, especially when considering reusing the dataset in a different setting or by different practitioners.

We see potential for future work that improves fairness and OOD generalization by leveraging powerful generative models but without explicitly relying on predefined categorical labels. When we consider synthetic images as an option for addressing performance gaps across subgroups, the following challenges still need to be addressed: reducing memorization for rare attributes and conditions; providing privacy guarantees; and accounting for unobserved characteristics.

## Methods

Our research complies with all relevant ethical regulations. We only repurposed existing assets and datasets and did not collect new assets for the purposes of our study, beyond annotations by dermatology experts for the generated images. The non-accessible data used in the study can be used for research purposes without further scrutiny or collection of consent from the source individuals.

### Datasets

In this section, we describe the datasets we used to train the downstream classifiers and diffusion models across the different modalities and medical contexts. Three different datasets were used, all of which are de-identified; informed consent was obtained from the participants in the original studies that collected these data.

#### Histopathology

We used data from the CAMELYON17 challenge^21^ that include labeled and unlabeled data from three different hospitals for training, as well as one in-distribution and one OOD validation hospitals. Data from the different hospitals differ because of the staining procedure used. The task was to estimate the presence of breast cancer metastases in the images, which are patches of whole-slide images of histological lymph node sections. The number of samples per hospital is given in Extended Data Table 1a; all subsets were approximately evenly split into those containing tumors and those that did not. We used the training data (302,436 examples) and the unlabeled data (1.8 million examples) to train the diffusion model. We performed patch-based instead of whole-slide classification to align with the WILDS challenge^22^ and follow-up works that evaluated methods on the same setup.

In terms of label distribution, there were 151,046 patches of healthy tissue in the training set and 151,390 patches of cancerous tissue. For the ID (validation) dataset, these statistics are 16,952 and 16,608, respectively, while in the OOD (validation) and OOD (test) splits there were 17,452 and 42,527 patches corresponding to each class, respectively (that is, both OOD datasets were perfectly balanced).

#### Chest radiology

**Extended Data Fig. 3:**
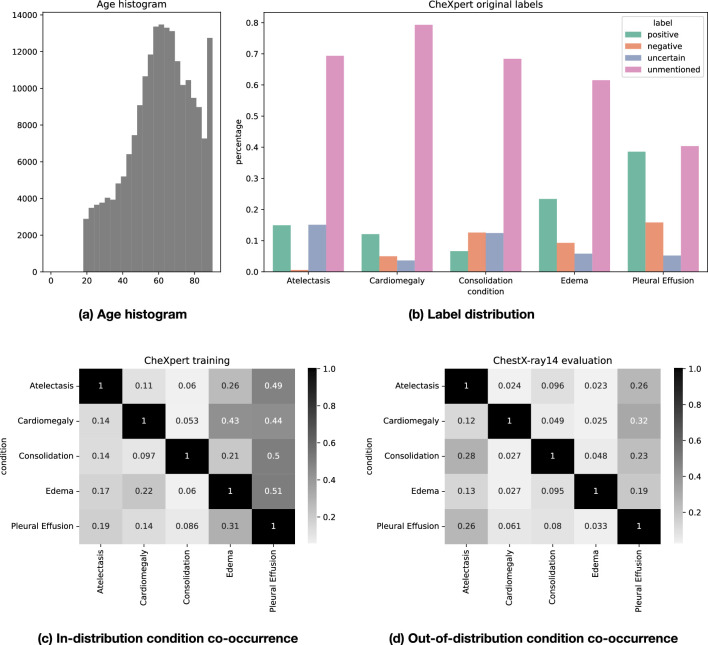
Chest radiology data statistics. Age histogram (a) and normalized label distributions (b) for the five conditions we consider in the CheXpert training dataset. Normalized co-occurrence of conditions in the CheXpert training (c) and the ChestX-ray14 evaluation (d) datasets. For each condition on the row *r* of each plot, the corresponding column *c* indicates the ratio of all samples with condition *r* that also have condition *c*. Note that more than two conditions can be present at once. We observe that in the training set it is much more common that more than one condition is present simultaneously.

We trained the cascaded diffusion and downstream discriminative model on a total of 201,055 samples from the CheXpert database^23^, with 119,352 individuals annotated as male and 81,703 as female (the dataset only contained binary gender labels). We show the age and original label distribution in Extended Data Fig. 3a,b. The original CheXpert training set contained positive, negative, uncertain and unmentioned labels. The uncertain samples were not considered when learning the diagnostic model, but they were used to train the diffusion model. The unmentioned label was considered a negative (that is, the condition was not present), which yielded a highly imbalanced dataset. The evaluation National Institutes of Health dataset^24^ denoted as OOD consisted of 17,723 individuals, out of which 10,228 were male and 7,495 were female.

Extended Data Fig. 3c,d illustrates how often different conditions co-occurred in the training and evaluation samples. Capturing the characteristics of a single condition can be challenging because they frequently coexist with other conditions in a single case. One characteristic example is pleural effusion, which was included in the diagnosis of atelectasis, consolidation and edema in approximately 50% of cases. However, the scenario is slightly different for the OOD ChestX-ray14 dataset, where for most pairs of conditions the corresponding ratio was much lower.

#### Dermatology

**Extended Data Fig. 4:**
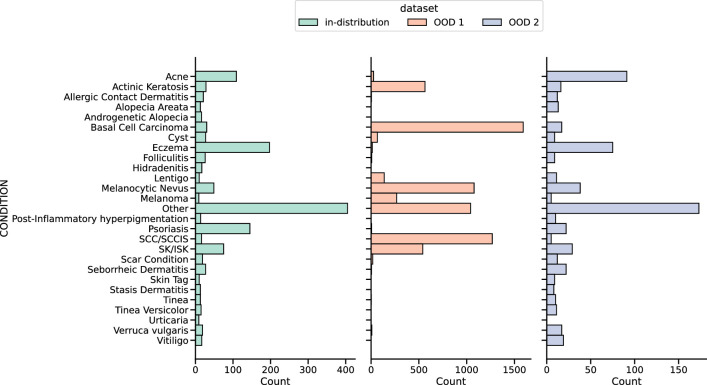
Dermatology data statistics. Condition distributions for in-distribution and out-of-distribution dermatology datasets. In-domain and OOD 2 distributions are much more similar in comparison to OOD 1. In particular, 3 out of 4 of the most prevalent conditions (that is acne, eczema and other) in the in-distribution dataset are also the most prevalent in OOD 2. However, there are only few examples of high-risk conditions like basal cell carcinoma and SCC/SCCIS, which are the two most prevalent conditions in OOD 1. We can see that overall the right-most dataset has a similar label distribution to the training dataset and, hence, is ‘less’ out-of-distribution than the other one.

The imaging samples in the dermatology dataset were often accompanied with metadata that include attributes like biological sex, age and skin tone. Skin tone was labeled according to the Fitzpatrick scale, giving rise to six categories (plus unknown). The ground truth labels for the condition were the result of aggregation of clinical assessments by multiple experts, who provided a list of top-3 conditions along with a confidence score (between 1 and 5). A weighted aggregate of these labels gave rise to soft labels that we used for training the generative and diagnostic models. The dermatology datasets were characterized by complex shifts with respect to each other as the label distribution, demographic distribution and capture process may all vary across them. To demonstrate the severity of the prevalence shift across locations, we visualized the distribution of conditions in the evaluation datasets in Extended Data Fig. 4.

To disentangle the effect of each of those shifts, we artificially skewed the source dataset along three sensitive attribute axes: sex, skin tone and age. Skewing the dataset allowed us to understand which methods performed better as the distribution shifts became more severe. For example, if our original dataset was skewed toward younger age groups, conditioning the generative model on age and (over)sampling from older ages could potentially help close the performance gap between younger and older populations. To study this aspect, we could not rebalance our datasets because we had too few samples from the long tail of our distribution with regard to the label or sensitive attribute. We skewed the training labeled dataset to make it progressively more biased (by removing instances from the least represented subgroups) and investigate how performance suffered because of skewing. For each sensitive attribute, we created new versions of the in-distribution dataset progressively more skewed to the high-data regions. We show how the resulting training dataset was skewed with respect to each of the sensitive attributes in Extended Data Table 1b–d. We also report similar demographic statistics for the three evaluation datasets in Extended Data Table 1e–g. The cascaded diffusion model was always trained on the union of the labeled training data and the total of unlabeled data across the three available domains. The discriminative model was always evaluated on the same three evaluation datasets (one in-distribution held-out dataset and two OOD datasets) for consistency.

### Related work

#### Learning augmentations with generative models in health

Generative models, especially generative adversarial networks (GANs)^29^, have been used by several studies to improve performance in different medical imaging tasks^30–34^ and, in particular, for underrepresented conditions^35^. Data obtained by exploring different latent image attributes through a generative model have also been shown to improve adversarial robustness of image classifiers^36^. In the clinical setting, GANs have been used by several studies to improve performance in different tasks, for example, disease diagnosis, in scenarios where few labeled samples were available. Such models have been used to augment medical images for liver lesion classification^30^, classification of diabetic retinopathy from fundus images^31^ and breast mass diagnosis in mammography^32^. In dermoscopic imaging^33^, a progressive generative model was introduced to produce realistic high-resolution synthetic images, while^34^ focused on improving balanced multiclass accuracy and, in particular, sensitivity for high-risk underrepresented diagnostic labels like melanoma^37^. It focused on a similar approach for chest X-rays by combining real and synthetic images generated with GANs to improve classifier accuracy for rare diseases^35^. It used conditional image generation in scenarios where the conditioning vector was not always available to disentangle image content and image style information. They applied the method to dermoscopic images (HAM10000 dataset) corresponding to seven types of skin lesions and lung computed tomography scans from the Lung Image Database Consortium-Image Database Resource Initiative.

Apart from whole-image downstream tasks, GAN-based augmentation techniques have been used to improve performance on pixel-wise classification tasks, for example, vessel contour segmentation on fundus images^38^ and brain lesion segmentation^39^. Given that pixel-wise downstream tasks were not within the scope of our study, we refer the reader to a more thorough review of GAN-based methods in medical image augmentation by Chen et al.^40^; Bissoto et al.^41^, in turn, provide an overview of GAN-based augmentation techniques with a main focus on skin lesion augmentation and anonymization.

Despite the wide variety of health applications that have adopted GAN-based generative models to produce learned augmentations of images, these are often characterized by limited diversity and quality^42^. More recently, DDPMs^19,20,43–45^ presented an outstanding performance in image generation tasks and have been probed for medical knowledge by Kather et al.^46^ in different medical domains. Other works extended diffusion models to three-dimensional magnetic resonance and computed tomography images^47^ and demonstrated that they can be conditioned on text prompts for chest X-ray generation^48^. Given the ethical questions around the use of synthetic images in medicine and healthcare^46,49^, it is important to make a distinction between using generative models to augment the original training dataset and replacing real images with synthetic ones, especially in the absence of privacy guarantees. None of these works claimed that the latter would be preferable, but rather came to the rescue when obtaining more abundant real data is either expensive or not feasible (for example, in the case of rare conditions), even if this solution is not a panacea^42^. While some studies view generative models as a means of replacing real data with ‘anonymized’ synthetic data, we abstain from such claims because greater care needs to be taken to ensure that generative models are trained with privacy guarantees, as shown by Carlini et al.^50^ and Somepalli et al.^51^.

#### Exploring fairness in health

Many scholars recently scrutinized ML systems and surfaced different types of biases that emerge through the ML pipeline, including problems due to data acquisition protocols, flawed human decision-making, missing features and label scarcity^52^. They identified and characterized various biases that can emerge during model development and are exacerbated during model deployment, and in clinical interactions, while they argued that ensuring fairness in those contexts is essential to advance health equity. The relevant literature discussed below was inspired by the realization that, if we break down performance of automated systems that rely on ML algorithms (for example, computer vision, judicial systems) based on certain demographic or socioeconomic traits, there can be vast discrepancies in predictive accuracy across these subgroups. This is alarming for applications influencing human life and it is particularly concerning in the context of computer-aided diagnosis and clinical decision-making.

One of the first studies to dive into the effect of training data composition on model performance across the sexes when using chest X-rays to diagnose thoracic diseases was the one led by Larrazabal et al.^12^. They found that the prevalence of a particular sex in the training set is directly linked to the predictive accuracy of the model for the same group at the test time. In other words, a model trained on a set highly skewed toward female patients would demonstrate higher accuracy for female patients at test time compared to a counterpart trained on a male-dominated set of images. Even though this finding might not come as a surprise, one would expect that a ML model used in clinical practice across geographical locations be robust to demographic shifts of this kind. In a similar vein, Seyyed-Kalantari et al.^13^ further explored how differences in age, race or ethnicity, and insurance type (as a proxy of socioeconomic status) are manifested in the performance of a classifier operating on chest radiographs. A crucial finding was that the algorithm would exhibit a higher false positive rate, that is, underdiagnose ethnic minorities. These effects were compounded for intersectional identities (that is, the false positive rate was higher for Black female patients compared to Black male patients). Similar findings were reported by Puyol-Antón et al.^53^ in a cardiac segmentation task with respect to sex and racial biases, and by Gianfrancesco et al.^54^ in a different modality (electronic health records) for patients with low socioeconomic status.

### Overview of methodology

The method is illustrated in Fig. 1b and leverages diffusion models to learn augmentations of the data. The approach consists of three main steps: (1) we trained a generative model given the available labeled and unlabeled data; (2) we sampled from the generative model according to a sampling strategy; (3) we enriched our original training dataset from the source (also called in-distribution) domain with the synthetic images sampled from the generative model and trained a diagnostic model (potentially for multiple labels, if more than one condition can be present at once). We treated the mixing ratio between real and synthetic as a hyperparameter in all three settings and we selected the best value based on model performance on the validation set. We provide more specific details about the experimental setting for each modality in the following section and the pseudocode for our method in Fig. 1a.

**Algorithm 1: pseudocode of proposed method**

Input: modality

 if Modality == "histopathology" then

   Num_labels ← 2

   A $$\in$$ {"hospital_id"}

 else if Modality == "radiology" then

   Num_labels ← 5

   A $$\in$$ {"sex", "race"}

 else if Modality == "dermatology" then

   Num_labels ← 27

   A $\in$ {"sex", "age", "skin_tone"}

 end if

Input: $${{X}}{{\in }}{{\mathbb{R}}}^{{\mathrm{Batch}}{}\times{\mathrm{Height}}{{\times }}{\mathrm{Width}}{{\times }}{\mathrm{Channels}}}{{;Y}}{{\in }}{{\mathbb{R}}}^{{\mathrm{Batch}}{{\times }}{{Nu}}{\mathrm{m}}\_labels}$$

 Train diffusion model $\hat{p}\sim {\mathrm{DDPM}}({{X}},Y,{{A}})$

 if Modality $\in$ {"radiology", "dermatology"} then

   Train upsampler diffusion model ${\hat{p}}_{\mathrm{upsampler}}\sim {\mathrm{DDPM}}({{X}},Y,{{A}})$

 end if

 Sample ${{X}}{\prime}$ from $\hat{p}$, ${\hat{p}}_{\mathrm{upsampler}}$ according to a fair distribution $\hat{p}(Y,{{A}})$

 We assume: $$\hat{p}({{A}})\sim \mathrm{uniform}$$, $$\hat{p}(Y)=p(Y)$$

Output: ${{X\text{'}}}{{\in }}{{\mathbb{R}}}^{{\mathrm{Samples}}{{\times }}{\mathrm{Height}}{{\times }}{\mathrm{Width}}{{\times }}{\mathrm{Channels}}}{\mathrm{;}}Y{{{\prime} }}{{\in }}{{\mathbb{R}}}^{{\mathrm{Samples}}{{\times }}{\mathrm{Nu}}{\mathrm{m}}\_labels}$ synthetic samples

 Sample random number ${rng}\in [\mathrm{0,1}]$

 Train diagnostic model $d({Y|}{{X}})=\mathrm{ResNet}({{X}})$ using ${{{x}}}_{d},{y}_{d}$ and mixing ratio $a$

 if $${rng} < a$$ then

   $${{{x}}}_{d},{y}_{d}\in {{(}}{{X}}{{,}}Y{{)}}$$

 else

   $${{{x}}}_{d},{y}_{d}\in {{(}}{{X\text{'}}}{{,}}Y{{{\prime} }}{{)}}$$

 end if

#### Experimental setting for each modality

##### Histopathology

For histopathology, we trained a diffusion model to generate images at 96 × 96 resolution, which is the smallest in comparison to the other imaging modalities. The data used to train the diffusion model consisted of labeled and unlabeled data only from the in-distribution hospitals. To condition the diffusion model, we considered either the diagnostic label (that is, cancer or no cancer) or the diagnostic label and hospital ID together. For the unlabeled data, which did not contain the diagnostic label, we padded the corresponding conditioning vector with zeros. We then sampled from the diffusion model assuming a uniform distribution across hospital IDs and preserving the diagnostic label distribution. The synthetic-to-real data ratio used in histopathology is 50:50, meaning that 50% of the total training samples corresponded to real patches and 50% to synthetic samples from the diffusion model. For the diagnostic model, we focused on a patch-based classification setup instead of whole-slide image classification. Both experimental design decisions, that is, the image resolution and the classification setup, were made to align with the WILDS challenge^22^ and the wealth of literature that evaluates ML methods on in-the-wild distribution shifts using the same setting^55^. We evaluated on the held-out in-distribution and OOD hospitals (results shown in Fig. 2).

##### Chest radiology

For chest radiology, we trained two diffusion models (one generating images at 64 × 64 resolution and one upsampling those generated images to 224 × 224 resolution) on labeled images from the in-distribution dataset. Therefore, in this scenario, we did not have access to any unlabeled data or data from the OOD dataset. This holds for both the diffusion models and the diagnostic model, that is, the OOD dataset was only used for evaluation. We conditioned both generative models on the diagnostic label only. While treating the synthetic-to-real data ratio as a hyperparameter, we found that training the downstream diagnostic model purely on synthetic data led to the best accuracy and fairness trade-off. We did not alter the diagnostic label distribution, that is, we used the labels of the real data to condition the diffusion models and yield a synthetic sample. In this setting, the model backbone was shared across all conditions, while a separate (binary classification) head was trained for each condition, given that multiple conditions can be present at once.

##### Dermatology

For dermatology, we trained two diffusion models (one generating images at 64 × 64 resolution and one upsampling those generated images to 256 × 256 resolution) on labeled images from the in-distribution dataset and unlabeled images from the in-distribution and OOD datasets. At no stage of training did we have access to labeled samples from the OOD datasets. We conditioned both generative models on the diagnostic label (padded with zeros for the unlabeled samples) or the diagnostic label and a demographic attribute. While treating the ratio of synthetic-to-real data as a hyperparameter, we found that training the downstream diagnostic model on 75% synthetic images and 25% real images yielded the best results. When we artificially skewed the dataset against certain demographic subgroups, we ensured that both the generative models and the diagnostic model had access to the same labeled examples (that is, we trained a different diffusion model for each skewed setting). When we sampled from the diffusion model, we preserved the diagnostic label distribution and assumed a uniform demographic attribute distribution.

#### Theoretical motivation

We motivated the use of generated data and demonstrated its utility in several toy settings, which simulate the problem of having only a few number of samples from the underlying distribution or parts of the underlying distribution. We wished to have high performance despite this lack of data. We demonstrated that even in these toy settings, synthetic data were useful.

We assumed we had a dataset ${D}_{\mathrm{train}}={\left\{\left({{{x}}}_{i},{y}_{i}{,{{a}}}_{i}\right)\right\}}_{i=1}^{N}$ where ${{{x}}}_{i},{y}_{i}$ is an image and label pair, ${{{a}}}_{i}$ is a list of attributes about the datapoint and 𝑁 is the number of training samples. The attributes may include attributes such as sex, skin type and age, or the hospital ID (in the case of histopathology). We had an additional dataset ${D}_{u}={\left\{{\hat{{{x}}}}_{j}\right\}}_{j=1}^{M}$ of unlabeled images, 𝑀 being the number of samples, that could be used as desired. We had a generative model $\hat{p}$ trained with ${D}_{\mathrm{train}}$ and ${D}_{u}$ (we make $\widetilde{\theta }$ implicit in the following). We dropped the subscripts in the following for simplicity where obvious.

To achieve fairness, we assumed we had a ‘fair’ dataset ${D}_{{\mathrm{f}}}={\left\{\left({{{x}}}_{i},{y}_{i}{,{{a}}}_{i}\right)\right\}}_{i=1}^{F}$ with 𝐹 datapoints that consisted of samples from the ‘fair’ distribution ${p}_{{\mathrm{f}}}$ over which we aimed to minimize the expectation of the loss. ${f}_{\theta }({{x}})$ was the classifier and $L$ the loss function (for example, binary cross-entropy). We aimed to optimize the following objective:

1 $$\mathop{{\bf{min }}}\limits_{{{\theta }}}\mathop{{\mathbb{E}}}\limits_{{{{D}}}_{{{{\mathrm{f}}}}}}\left({{L}}\left(\;{f}_{{{\theta }}}({{x}}),y,{{a}}\right)\right)$$

We can decompose the data generating process into ${p}_{{\mathrm{f}}}({{x}}|{{a}},y){p}_{{\mathrm{f}}}({{a}}{|y}){p}_{{\mathrm{f}}}(\;y)$. For example, we may have created ${D}_{{\mathrm{f}}}$ by sampling uniformly over an attribute (such as sex) and labels. We assumed that the training dataset ${D}_{\mathrm{train}}{\subset D}_{{\mathrm{f}}}$ was sampled from a distribution ${p}_{\mathrm{train}}$ where ${p}_{\mathrm{train}}({{x}}|{{a}},y){=p}_{{\mathrm{f}}}({{x}}|{{a}},y)$. When ${p}_{\mathrm{train}}(\;y,{{a}}){\ne p}_{{\mathrm{f}}}(\;y,{{a}})$, then we have a distribution shift between the training and fair distribution (for example, the training distribution is more likely to generate images of a particular attribute or combinations of label and attribute than the fair distribution).

We aimed to combine the training dataset ${D}_{\mathrm{train}}$ and synthetic data sampled from the generative model $\hat{p}$ to mimic most closely the fair distribution and improve fairness. We constructed a new dataset ${\hat{D}}$ according to a distribution ${\hat{p}}$ from these distributions using some probability parameter $\alpha$:

2 $$\left({{x}},{{a}},y\right) \sim{p^{\prime}}\left\{\begin{array}{l}\left({{x}},{{a}},y\right) \sim {D}_{\mathrm{train}}\qquad:\alpha \\ \left({{x}},{{a}},y\right),{x}\sim \hat{p}\left({{x}}|y,{{a}}\right),\left({{a}},y\right) \sim \hat{p}({{a}}{{,}}y)\qquad:(1-\alpha )\end{array}\right.$$

So instead of minimizing equation (1), we minimized the following sum of expectations:

3 $$\mathop{{\bf{min }}}\limits_{{{\theta }}}\alpha \mathop{{\mathbb{E}}}\limits_{\left({{x}},{{a}},y\right) \sim {D}_{{{\mathrm{train}}}}}\left({{L}}\left(\;{f}_{{{\theta }}}({{x}}),{{a}},y\right)\right)+(1-\alpha )\mathop{{\mathbb{E}}}\limits_{\left({{x}},{{a}},y\right) \sim \hat{p}}\left({{L}}\left(\;{f}_{{{\theta }}}({{x}}),{{a}},y\right)\right)$$

The question is then how to choose $\alpha$ and $\hat{p}({{a}},y)$. For all settings in the main article, we maintained the label distribution $\hat{p}(\;y)=p(\;y)$ but sampled uniformly over the attribute $\alpha$. We validated this choice on dermatology in the Supplementary Information. We treated $\alpha$ as a hyperparameter in all settings.

### Models

#### Upsampler preprocessing

Whenever we required an upsampler (that is, in radiology and dermatology), we trained it by preprocessing the original images using the following steps: (1) upsampled images from the 64 × 64 input resolution to the desired output resolution with bilinear interpolation and used an anti-alias with 0.5 probability; (2) added random Gaussian noise with 0.2 probability and *σ* = 4.0 (in the (0–255) range); (3) applied random Gaussian blurring with a 7 × 7 kernel and *σ*_mean_ = 0, *σ*_s.d._ = 0.2; (4) quantized the image to 256 bins; and (5) normalized the image to the (−1 to 1) range.

#### Dealing with missing labels

For both the generative model and the upsampler, we filled the conditioning vectors with zeros (indicating an invalid vector) for the unlabeled data. This allowed us to use classifier-free guidance^20^ to make images more ‘canonical’ with respect to a given label or property.

In this section, we describe the exact model architecture used for the trained diffusion models and classifiers, as well as the hyperparameters used for the presented results. Hyperparameters were selected based on the baseline model performance on the respective in-distribution validation sets and held constant for the remaining methods. This meant that we did not finetune hyperparameters for each method (other than the baseline) separately. We use the DDPM as presented by refs. ^19,20,43^ for the generation and the upsampler (only the radiology and dermatology datasets required higher-resolution images). The backbone model was always a UNet architecture. The hyperparameters used for the cascaded diffusion models were based on the standard values mentioned in the literature with minimal modifications. We present all hyperparameters in Extended Data Table 2.

#### Standard augmentations

##### Histopathology

For this modality, augmentations included brightness, contrast, saturation and hue jitter. Hue and saturation were sufficient to achieve the high-quality results described by Tellez et al.^56^.

##### Chest radiology

The heuristic augmentations considered for this modality included: random horizontal flipping; random cropping to 202 × 202 resolution; resizing to 224 × 224 with bilinear interpolation and anti-alias; random rotation by 15 degrees, shifting luminance by a value sampled uniformly from the (−0.1 to 0.1) range; and shifting contrast using a value uniformly sampled from the (0.8 to 1.2) range (that is, pixel values were multiplied by the shift value and clipped to remain within the (0 to 1) range).

##### Dermatology

For this modality, we used the following heuristic augmentations: random horizontal and vertical flipping; adjusting image brightness by a random factor (maximum $\delta =0.1$); adjusting image saturation by a random factor (within the (0.8 to 1.2) range); adjusting the hue by a random factor (maximum $\delta =0.02$); adjusting image contrast by a random factor (within the (0.8 to 1.2) range); random rotation within the (−150 to 150) range; and random Gaussian blurring with standard deviation uniformly sampled from the following values: {0.001, 0.01, 0.1, 1.0, 3.0, 5.0, 7.0}.

#### Baselines

In all contexts, we considered the strongest heuristic augmentations as a baseline. These augmentations (heuristic or learned) can be combined with any alternative learning algorithm that aims to improve model generalization. For the sake of our experiments, we used empirical risk minimization^57^ because there is no single method that consistently outperforms it under distribution shifts^55^. Even though our experiments and analysis focus on DDPMs for generation, any conditional generative model that produces high-quality and diverse samples can be used. In general, the risk, that is, how well the algorithm will fit the data, cannot be computed on the true data distribution $P(x,y)$ because it is unknown to the learning algorithm. However, we could compute an approximation, called empirical risk, by averaging the loss function on the training set samples.

##### Histopathology

For this modality, all models used the same ResNet-152 backbone. We compared (1) a baseline using no augmentation (Baseline) and (2) one using standard color augmentations (Color augm.) as applied in standard ImageNet training. This augmentation included brightness, contrast, saturation and hue jitter. Hue and saturation were sufficient augmentations to achieve the highest-quality results by Tellez et al.^56^; hence, we did not evaluate other heuristic augmentations. Our baseline did not use pretraining because it previously did not yield any benefits on this particular dataset as reported by Wiles et al.^55^. We also compared the models to those applying heuristic color augmentations on top of the synthetic data.

##### Chest radiology

All models used the same BiT-ResNet-152 backbone^58^. We considered baselines that use (1) different pretraining, (2) different heuristic augmentations and combinations thereof, and (3) focal loss. We investigated using JFT^59^ and ImageNet-21K^60^ for pretraining to explore how much different pretraining datasets impacted the final results. We investigated using RandAugment^61^, ImageNet Augmentations as described above, and RandAugment + ImageNet Augmentations to determine how much performance we could gain by using heuristic augmentations. Finally, we considered using focal loss^62^, which was developed to improve performance on imbalanced datasets.

##### Dermatology

All models used the same BiT-ResNet backbone^58^. We considered baselines that (1) used different pretraining, (2) used different heuristic augmentations, (3) resampled the dataset and (4) used the focal loss. We investigated using JFT^59^ and ImageNet-21K^60^ for pretraining. We investigated using RandAugment^61^, ImageNet Augmentations and RandAugment + ImageNet Augmentations. We then resampled the dataset so that the distribution over attributes was even (we upsampled samples from low-data regions so that they occurred more frequently in the dataset). Finally, we considered using focal loss^62^, which was developed to improve performance on imbalanced datasets.

### Evaluation details

#### Experimental setup

To account for potential variations with respect to model initialization, we evaluated all versions of our model and baselines with five different initialization seeds and report the average and standard deviation across those runs for all metrics. We ran all experiments on tensor processing units.

#### Fairness metrics

Different definitions of fairness have been proposed in the literature, which are often at odds with each other^63^. In this section we discuss our choice of fairness metrics for each modality. In histopathology, we used the gap between the best and worst performance among the in-distribution hospitals. For radiology, we considered AUC parity, namely the parity of the area under the ROC for different demographic subgroups identified by the sensitive attribute $A$, which can be seen as the analog of equality of accuracy^64^. Therefore, for this modality, we report the AUC gap between males and females in Fig. 3a. We considered this most relevant given that the positive and negative ratio of samples across all conditions was very imbalanced.

In dermatology, we report the gap between the best and worst subgroup performance, where subgroups are defined based on the sensitive attribute axis under consideration in Fig. 4. We also report the central best estimate for the a posteriori estimate of performance (that is, top-3) difference between a group and its outgroup. The steps to obtain the values plotted in Supplementary Fig. 7 are the following: (1) we defined a group (and its matching outgroup) as the set of instances characterized with a particular value of a sensitive attribute *A* = *α*, that is, group = {(*xi,ci*)|*ai* = *α*} and group = {(*xi,ci*)|*ai* ≠ *α*}. Here *A* ⊆ {sex, skin type, age}; (2) we assumed a uniform Beta distribution *Beta*(1,1) as a prior for the performance difference between top_3_^group^ and top_3_^outgroup^ and fitted this to the observed data; (3) we sampled *n* = 100,000 samples from the estimated posterior differences between tôp_3_^group^ and tôp_3_^outgroup^ and report the spread, that is, the standard deviation of the maximum a posteriori estimates, which can be interpreted as the central best estimate for fairness.

#### Setup for distribution shift estimation

We computed domain mismatches considering the space where decisions are performed, that is, the output of the penultimate layer of each model. Thus, we projected each data point from the input space of size ${{\mathfrak{R}}}^{64x64}$ to a representation of size ${{\mathfrak{R}}}^{6144}$ and then computed the maximum mean discrepancy (MMD) between two distributions (that is, datasets). Given two distributions $U$ and $Z$, their respective samples $\hat{U}=\{{u}_{1},\ldots ,{u}_{N}\}$ and $\hat{Z}=\{{z}_{1},\ldots ,{z}_{N}\}$, and a kernel $K$, we considered the MMD empirical estimate as defined below:

4 $$\begin{array}{l}{\widehat{{\rm{MMD}}}}^{2}(u,{\mathcal{Z}})=\frac{1}{N(N-1)}\mathop{\sum }\limits_{i,\;j=1}^{N}K({u}_{i},{u}_{j})+\frac{1}{N(N-1)}\mathop{\sum }\limits_{i,\;j=1}^{N}K({z}_{i},{z}_{j})\\\qquad\qquad\qquad\quad-\frac{2}{{N}^{2}}\mathop{\sum }\limits_{i,\;j=1}^{N}K({u}_{i},{z}_{j})\end{array}$$

We used a cubic polynomial kernel to minimize the number of hyperparameters to be selected and to capture mismatches between up to the third-order moments of each distribution. We computed $S=30$ estimates of MMD between all pairs of domains using representations from the different models considering samples of size $n=300$. A Mann–Whitney *U*-test under a significance level of 95% was then carried out to test for the hypothesis that, for a fixed pair of distributions, the data augmentation strategy had a significant effect on the estimated MMD values. Importantly, we highlight that models were trained under the same experimental conditions so that our analysis was capable of isolating the effect of the data augmentation protocol on the estimated pairwise distribution shifts.

### In-depth analysis for dermatology

In this section, our analysis focuses on the modality of dermatology and puts forward several properties of our synthetic data that may be important for our experimental results, which demonstrate the utility of synthetic data for improving performance.

#### Generated images are diverse

First, we show images generated at high resolution for this challenging natural setting and several dermatological conditions in Fig. 5. Our conditional generative model captured the characteristics well for multiple, diverse conditions, even for cases that are more scarce in the dataset, such as seborrheic dermatitis, alopecia areata and hidradenitis.

#### Generated images are realistic

We further evaluated how realistic the generated images were as determined by expert dermatologists to validate that these images did contain properties of the disease used for conditioning. Synthetic images did not need to be perfect, as we were interested in the downstream diagnostic performance. However, being able to generate realistic images validates that the generative model captures the relevant features of the conditions. To evaluate this, we asked dermatologists to rate a total of 488 synthetic images each, evenly sampled from the four most common classes (eczema, psoriasis, acne, seborrheic keratosis/irritated seborrheic keratosis) and four high-risk classes (melanoma, basal cell carcinoma, urticaria, SCC/SCCIS). They were tasked to first determine if the image was of a sufficient quality to provide a diagnosis. They were then asked to provide up to three diagnoses from over 20,000 common conditions with an associated confidence score (out of 5, where 5 was most confident). These 20,000 conditions were mapped to the 27 classes we used in this paper (one class, Other, encompasses all conditions not represented in the other 26 classes). We report the mean and standard deviation for all metrics across the three raters; 50.0 ± 12.6% of those images were of a sufficient quality for diagnosis, while dermatologists had an average confidence of 4.13 ± 0.43 out of 5 for their top diagnosis. They had a top-1 accuracy of 56.0 ± 11.9% on the generated images and a top-3 accuracy of 67.7 ± 12.5%.

We compared these numbers to a set of real images of the same eight conditions considered above (for the images considered, most raters considered the diagnosis of this disease as the most prevalent in the image). Among 101 board-certified dermatologists rating 789 real images in total, we found that their top-1 accuracy was 54.0 ± 21.1% and top-3 accuracy 67.1 ± 22.7%; a slightly higher performance in terms of top-1 (63%) and top-3 (75%) accuracy was shown by Liu et al.^4^ across a more diverse set of dermatological conditions. For this latter analysis, if an image was rated by *n* dermatologists, we considered a single rater’s accuracy with respect to the aggregated diagnosis of the remaining *n* *−* *1* raters. This demonstrates that, when diagnosable as per the experts’ evaluation, synthetic images are indeed representative of the condition they are expected to capture and similarly so to the real images. Even though not all generated images were diagnosable, this can also be the case for real samples, given that the images used to train the generative model did not necessarily include the body part or view that best reflected the condition.

#### Generated images are canonical

**Extended Data Fig. 5:**
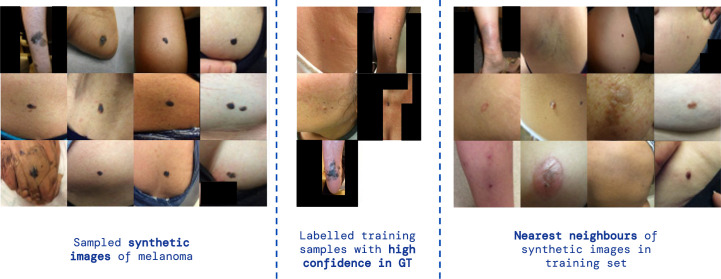
Generated images in dermatology are canonical examples of the condition. (*Left*) The diffusion model can produce an infinite amount of synthetic images for a particular condition that is inherently more scarce (we have fewer than 50 samples of melanoma in our training dataset). (*Middle*) The images that experts have identified with melanoma with high confidence (a combination of individual’s confidence in diagnosis and expert consensus). (*Right*) The nearest neighbours from the training samples identified for each of the synthetic images based on *l*-norm in pixel space.

We hypothesized that the reason why models are more robust to prevalence shifts is because of synthetic images being more canonical examples of the conditions. To understand how canonical ground truth images for a particular condition were, we investigated cases with a high degree of concordance in raters’ assessments and compared those to synthetic images for the same condition. More specifically, we thresholded the aggregated ground truth values to filter the images within the training data that experts were most confident about presenting as a condition. The aggregation function operates as follows: assume we have a set of four conditions $\{A,{B},{C},{D}\}$; if rater ${R}_{1}$ provides the following sequence of $(\mathrm{condition},\mathrm{confidence})$ diagnosis tuples $\{(A,4),(B,3)\}$ and rater ${R}_{2}$ provides $\{(A,3),(D,4)\}$, then we obtained the following soft labels $\{0.5,\mathrm{0.167,0,0.333}\}$ (after weighting each condition with the inverse of its rank for each labeler, summing across labelers and normalizing their scores to 1). If we looked for instances for which there is consensus among raters and high confidence that a condition is present, we could threshold the corresponding soft label for that condition with a strict threshold, for example, $t=0.9$. In our example, this did not hold for any of the four conditions; however, if we lowered the threshold to 0.5, then it would hold for condition $A$. In Extended Data Fig. 5 we show an example for melanoma. For this particular diagnostic class, we generated multiple synthetic instances of the condition, while we recovered only five images (out of more than 15,000) that clinicians rated with high confidence, that is, ${t}_{\mathrm{melanoma}}=0.9$. The nearest neighbors from the training dataset identified based on an ${l}^{2}$-norm are also shown in Extended Data Fig. 5.

#### Generated images are better at aligning feature distributions

Previous work on OOD generalization^65–67^ pointed out that several factors can affect the performance of a model on samples from domains beyond the training data. In this analysis, we investigated the models trained with our proposed learned augmentations in terms of changes in distribution alignment between all pairs of distributions measured using MMD^68^. We computed domain mismatches considering the space where decisions are performed and projected each data point from the input space to a representation. We found that learned augmentations yielded on average 18.6% lower MMD compared to heuristic augmentations (for more details, refer to Methods, ‘Distribution shift estimation’) which leads to the following conclusions: (1) data augmentation has a significant effect on distribution alignment. Improvement on OOD performance suggests this is happening via learning better predictive features rather than capturing spurious correlations; (2) the generated data help the model to better match different domains by attenuating the overall discrepancy between domains; (3) given the minor decline in performance when adding generated data in the less skewed setting, as shown in Fig. 4, these findings suggest that learning such features might conflict with learning spurious correlations that were helpful for in-distribution performance. In other words, introducing synthetic data allowed the diagnostic model to allocate more capacity for disease-specific features rather than domain-specific (for example, hospital) features.

#### Synthetic images reduce spurious correlations

To further compare the effect of different augmentation schemes on the features learned by the diagnostic model, we investigated the representation space occupied by all considered datasets, including samples obtained from the generative model. In practice, we projected *n* randomly sampled instances from each dataset to the feature space learned by each model and applied the principal component analysis algorithm^69^ to identify the most significant modes of variation. We then extracted the number of principal components required to represent different fractions of the variance across all instances. We observed that for a fixed dataset, features from models trained with synthetic data require 5.4% fewer principal components to retain 90% of the variance in the latent feature space (results for different fractions are provided in Supplementary Fig. 3). This indicates that using synthetic data induces more compressed representations compared to augmenting the training data in a heuristic manner. Considering this finding in the context of the results in Extended Data Table 3, we posit that the observed effect is due to domain-specific information being attenuated in the feature space learned by models trained with synthetic data. This suggests that our proposed approach is capable of reducing the model’s reliance on correlations between inputs and labels that do not generalize OOD. For example, if most images of melanoma in the training set correspond to individuals with light skin tones, the model could learn to predict skin tone instead of the condition.

### Additional results

#### Histopathology

##### Generated samples

Extended Data Fig. 2 presents some examples of generated images by the class-conditioned diffusion models for healthy and abnormal whole-slide images of histological lymph node sections.

##### Label efficiency

The histopathology dataset was balanced, so it did not demonstrate whether synthetic data were useful in the presence of data imbalance. To understand the impact of the number of labeled examples on both in-distribution and OOD generalization, we created different variants of the labeled training set, where we varied the number *n* of samples from two of the training hospitals. The number of labeled examples from one hospital was constant. For each value of *n*, we trained a diffusion model using the labeled and unlabeled dataset. We considered two settings when conditioning the diffusion model: (1) we used only the diagnostic label when available; and (2) we used the diagnostic label together with the hospital ID.

We subsequently sampled synthetic samples from the diffusion model and trained a downstream classifier that we evaluated on the held-out in-distribution and OOD datasets.

We trained the downstream classifier with five seeds and plotted the mean and standard deviation in Extended Data Fig. 1a. We found that using synthetic data outperformed both baselines consistently over varying *n* in-distribution. The same holds for the low-data regime in the OOD setting. Using our approach can achieve the performance that the baseline model achieves with 1,000 labeled samples in-distribution using only 1–10 samples (yielding 3× better label efficiency in terms of the low-data regions). We also performed color augmentation on top of the generated samples and found that this generalized best overall, leading to approximately 5% improvement OOD over the model trained with color augmentations in the high-data regime (1,000–10,000 samples) and approximately 4.3% in the low-data regime (one labeled sample).

#### Chest radiology

##### Generated samples

Extended Data Fig. 2 presents examples of the images generated by the class-conditioned diffusion models for healthy chest X-rays and those with thoracic conditions. Higher-resolution images were generated for chest X-rays (224 × 224) compared to histopathology (96 × 96), which requires training a separate upsampler diffusion model in the former case.

##### Results per condition

We show the model’s AUC values across method in-distribution and OOD in Extended Data Fig. 1b. Some conditions, that is, cardiomegaly, benefited significantly from synthetic data, while others, for example, effusion, benefited more from OOD than in-distribution. Finally, for atelectasis, synthetic images were only marginally beneficial to OOD.

##### Results for race

We use the primary race labels obtained from https://stanfordaimi.azurewebsites.net/datasets/192ada7c-4d43-466e-b8bb-b81992bb80cf for the in-distribution CheXpert dataset. We plotted the difference between the best and worst performing group in terms of ROC-AUC against overall performance across conditions in Fig. 3b. The number of individuals associated with each racial label was as follows: white, 6,047; other, 1,623; white, non-Hispanic, 1,359; Asian, 1,254; unknown, 1,019; Black or African American, 557; race and ethnicity unknown, 513; other, Hispanic, 239; native Hawaiian or other Pacific Islander, 177; Asian, non-Hispanic, 166; Black, non-Hispanic, 133; white, Hispanic, 63; other, non-Hispanic, 39; patient refused, 31; American Indian or Alaska native, 30.

#### Dermatology

##### Multiple metrics across datasets

For each sensitive attribute and distribution shift, we ran all baselines with five random seeds. We then trained a diffusion model at 64 × 64 (for faster iteration) using the labeled and unlabeled data for that specific shift and combined synthetic and real data. We considered conditioning either only on the label or on the label and sensitive attribute. We plot the top-3 accuracy, balanced accuracy, fairness metric and high-risk sensitivity on the in-distribution and OOD datasets in Supplementary Figs. 5–8. For both accuracy and fairness, we plotted the normalized metric. (We plotted the improvement over the baseline, where we use Pretrained on JFT as the baseline.)

First, we discuss the results on the accuracy metrics. Across all distribution shifts and all datasets, using generated data either improved or maintained the accuracy metrics on dermatology. In particular, generated data seemed to help most on the OOD, which had a stronger prevalence shift with respect to the training set and on the balanced accuracy metric.

Using heuristic augmentation helped, in particular RandAugment, which consistently improved over the baseline. The other methods (oversampling and focal loss) gave minimal improvements.

Next, we investigated results on the fairness metrics in Supplementary Fig. 7. Using heuristic augmentation led to no consistent improvement over the baseline. However, for sex, skin tone and age, our approach of using generated data consistently improved on or maintained the performance of the baseline model. This was true even on the OOD datasets, but more so for those characterized by stronger shifts in comparison to the in-distribution dataset (that is, OOD 2 was much more similar to the in-distribution dataset compared to OOD 1, where we observed the strongest improvements). This is impressive as Schrouff et al.^18^ demonstrated that improving fairness on in-distribution datasets does not guarantee performance improvements on OOD datasets. (Note that there were no skin tone labels for the OOD datasets, so for skin tone we only report the results on the in-distribution dataset.)

Finally, we investigated how using synthetic data impacts high-risk sensitivity in Supplementary Fig. 8. In the diagnostics, it is imperative not to miss someone with a high-risk condition. Thus, we investigated whether using synthetic data negatively or positively impacted the model’s ability to correctly identify the images of a high-risk condition. Of the 27 classes, three were identified as high-risk conditions: basal cell carcinoma, melanoma and SCC/SCCIS. By adding additional data, we wanted to improve (or at least not harm) high-risk sensitivity. We investigated high-risk sensitivity on both the training dataset (held out part of it) and the two OOD datasets. We found that across distribution shifts and datasets, using the additional synthetic data either maintained or improved high-risk sensitivity, most notably on the most OOD dataset. Moreover, synthetic data were consistently similar or better than heuristic augmentation on this metric.

We found that in dermatology, using synthetic data had a host of benefits. While it can to some extent improve balanced accuracy while maintaining overall accuracy, additional synthetic data can improve fairness metrics both in-distribution and OOD and high-risk sensitivity for both in-distribution and OOD datasets. This demonstrates that using synthetic data as an augmentation tool has promise for improving fairness and the diagnosis of high-risk conditions.

##### Distribution shift estimation

We computed domain mismatches considering the space where decisions are performed, that is, the output of the penultimate layer of each model. Thus, we projected each data point from the input space to a representation. We computed multiple estimates ($S$) of MMD between all pairs of domains using representations from the different models considering samples of size $n$. Models were trained under the same experimental conditions so that our analysis was capable of isolating the effect of data augmentation on the estimated pairwise distribution shifts. In addition to the heuristic augmentation discussed in the main text, we further included models trained with RandAugment in this analysis. All findings are summarized in Extended Data Table 3.

From the three considered augmentation schemata, RandAugment yielded representations that were more aligned in comparison to the learned and heuristic augmentations for all pairs of domains. We hypothesized this augmentation strategy would promote better in-distribution generalization by allowing domain-specific cues to be removed at the expense of learning spurious correlations. Evidence to support this hypothesis can be found in Supplementary Fig. 7, which shows that models trained with RandAugment yielded improved performance in-distribution and in the OOD 2 domain, which is more similar to the training distribution than OOD 1 (Extended Data Fig. 4).

##### Individuals underserved by models

**Extended Data Fig. 6:**
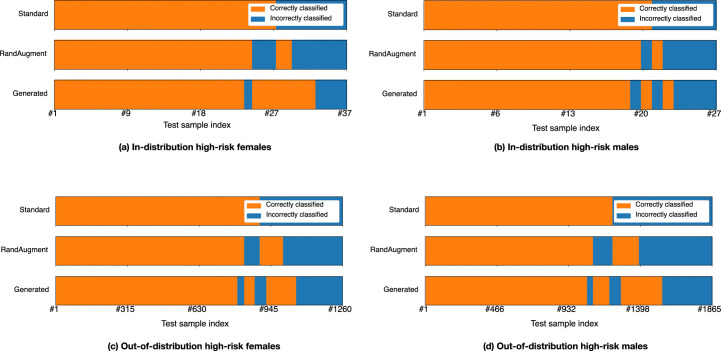
Misclassification rates for different subgroups. Mis-classification analysis on individuals with high-risk conditions depending on their sex attribute. We investigate the impact of a skewed training dataset with respect to the underrepresented sex on correctly detecting high-risk conditions for that subgroup in- and out-of-distribution. In each plot, we show the test sample index on the x-axis to investigate which individuals are consistently misclassified by all approaches. Sample indices are re-ordered such as to form contiguous blocks of correctly and incorrectly classified samples. It is worth noting that the source domain is more skewed towards females (see Table A1 for details).

Inspired by a recent study by Bommasani et al.^70^ that looked at how often the same individuals are underserved by ML models that have been trained on the same data, we investigated whether the same individuals with high-risk conditions were consistently misclassified. In Extended Data Fig. 6, we illustrate for all sample IDs across the in-distribution and OOD evaluation datasets whether there were particular individuals within each demographic subgroup (male or female) who benefited more from the generated data than from other augmentation techniques. For each of the three setups, that is, (1) standard ImageNet augmentations, (2) RandAugment and (3) generated data, we performed five training runs and considered a test sample as incorrectly classified for a setup if it had been consistently misclassified by its five trained models. For better comparison, we reordered the sample indices such as to form contiguous blocks of correctly and incorrectly classified samples. While most of the individual predictions were the same between setups, each setup enabled some samples to be correctly classified, which the other setups could not. Particularly, in Extended Data Fig. 6a, d, training with generated data significantly reduced the number of consistently misclassified samples compared to standard ImageNet augmentations or RandAugment. Even though the training dataset was more skewed toward females, OOD males with high-risk conditions in panel d were more often correctly classified for a model trained with the generated data. Hence, using generated data reduced the number of underserved individuals compared to standard augmentation techniques, which only applied basic transforms to the original data. Finally, we observed that these training setups were complementary as each of them had its own set of well-classified samples. This could open new research directions for model ensembling to create new models that would benefit from this diversity in individual predictions.

**Table 1:** Summary of the experimental setup and major improvements for each modality

Experimental setup		Histopathology	Radiology	Dermatology
Diffusion model	Conditioning variable	Diagnosis and hospital ID	Diagnosis	Diagnosis and demographic attribute
	Access to unlabeled data (additional to the diagnostic model’s training data)	Yes	No	Yes
	Using OOD unlabeled data	No	No	Yes
Diagnostic model	Synthetic/real data ratio	50:50	100:0	75:25
	Performance metric	Top-1 accuracy	ROC-AUC	High-risk sensitivity
	Relative performance improvement with regard to baseline without augmentations	48.5%	5.2%	27.3%
	Absolute fairness improvement with regard to baseline	↓ 30.0% in-distribution	↓ 0.031 OOD	↓ 0.044 OOD

Performance improvements are reported with respect to the respective baseline method, while fairness improvements are reported in absolute terms with respect to the baseline for the corresponding performance metric.

### Reporting summary

Further information on research design is available in the Nature Portfolio Reporting Summary linked to this article.

## Online content

Any methods, additional references, Nature Portfolio reporting summaries, source data, extended data, supplementary information, acknowledgements, peer review information; details of author contributions and competing interests; and statements of data and code availability are available at 10.1038/s41591-024-02838-6.

### Supplementary information

Supplementary Information Results in simplified settings, principal component analysis for spurious correlations, discussion on the sampling schemes and additional results for the dermatology setting.

Reporting Summary

## Figures and Tables

**Extended Data Table 1:**
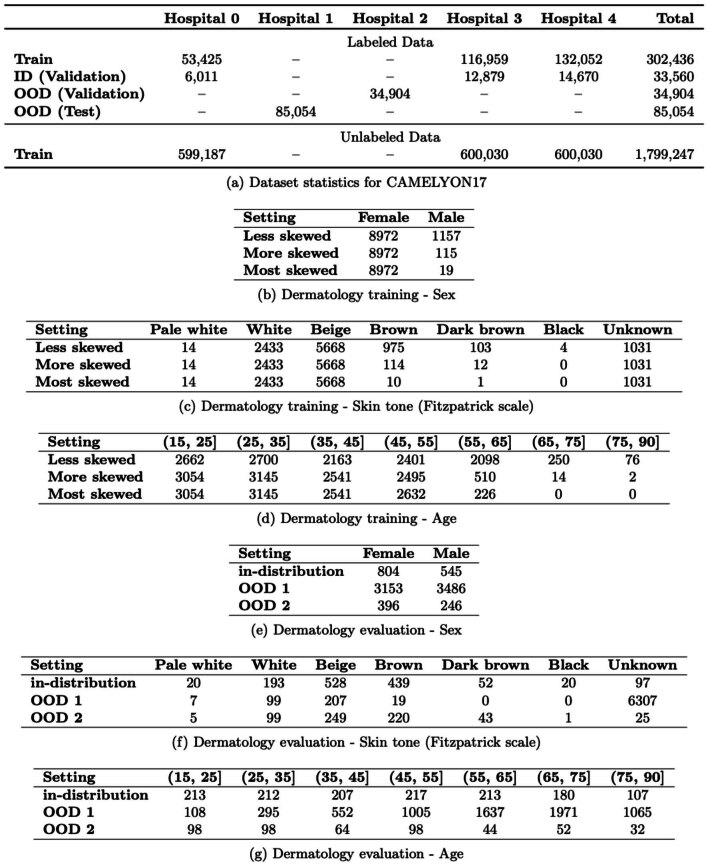
Dataset statistics for histopathology and dermatology

**a**, CAMELYON17 dataset statistics. For all hospitals, the labeled data are approximately evenly split between tumorous and nontumorous images. **b**–**g**, Number of training (**b**–**d**) and evaluation (**e**–**g**) samples annotated with the corresponding sensitive attribute label across the different splits.

**Extended Data Table 2:**
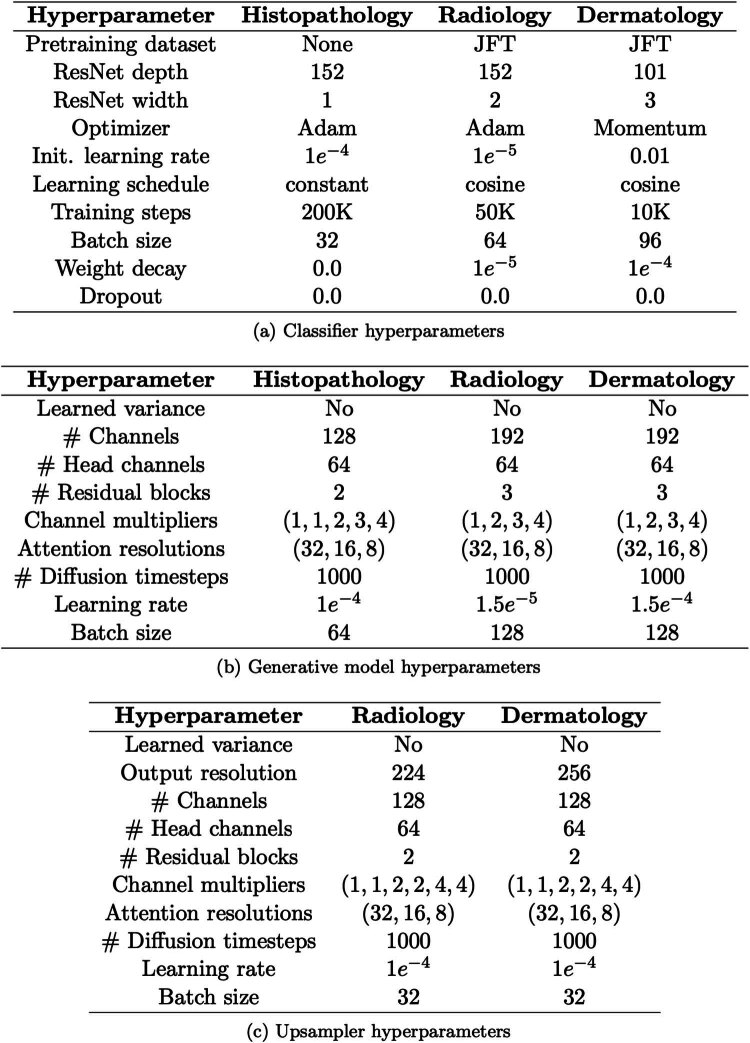
Model hyperparameters

Model hyperparameters for classifier and generative (low-resolution and upsampler) models.

**Extended Data Table 3:**
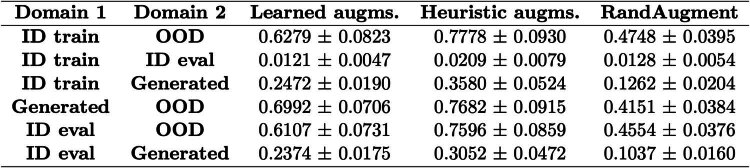
Distribution shift estimation

Maximum mean discrepancy values between pairs of domain distributions with learned and heuristic augmentations. ID, in-distribution; OOD, out of distribution. Differences for all comparisons are statistically significant based on a Mann–Whitney *U*-test with a significance level of 95%.

## Data Availability

The de-identified teledermatology data used in this study are not publicly available due to restrictions in the data sharing agreement. The data are available for noncommercial purposes for an administrative fee, provided that the requesting entity can comply with applicable laws and the privacy policy of the data provider. Contact dermatology-research@google.com, who can help forward any requests to the source, with a maximum response time frame of 2 weeks. Data used in the training and evaluation of chest radiology classification, including CheXpert and ChestX-ray14, are publicly available. Data used for in-distribution fine-tuning and evaluation of pathology metastasis detection are publicly available on the CAMELYON challenge website. Moreover, ImageNet-21K^71^ and JFT-300M^59^ have been used to pretrain the baseline supervised models. ImageNet-21K is publicly available at the ImageNet website (https://www.image-net.org/), but the JFT-300M dataset is not publicly available due to restrictions in the data sharing agreement.

## Code availability

Several major components of our work are available in open source repositories, such as the Haiku library. The codebase and pretrained weights for the BiT models are available at the Big Transfer GitHub (https://github.com/google-research/big_transfer)^72^. The guided diffusion implementation is based on the Diffusion Github repository (https://github.com/hojonathanho/diffusion/blob/1e0dceb3b3495bbe19116a5e1b3596cd0706c543/diffusion_tf/diffusion_utils_2.py)^73^. All experiments and implementation details are described in sufficient detail in the Methods to support replication with nonproprietary libraries. We have provided model weights for generative models trained on the public CAMELYON dataset at https://github.com/google-deepmind/augmentations_medical_images (ref. ^74^), along with an inference code to reproduce the final results using these models. Other components used and developed for this study cannot be shared publicly because of their proprietary nature.
