# Supplementary material for: Generative models improve fairness of medical classifiers under distribution shifts
Source: Nat Med. 2024 Apr 10;30(4):1166–73. doi: 10.1038/s41591-024-02838-6 (PMC11031395; doi:10.1038/s41591-024-02838-6)
Supplement: Supplementary file 1 — Results in simplified settings, principal component analysis for spurious correlations, discussion on the sampling schemes and additional results for the dermatology setting. [file 41591_2024_2838_MOESM1_ESM.pdf]

nature medicine

Article https://doi.org/10.1038/s41591-024-02838-6

Generative models improve fairness of medical classifiers under distribution shifts

In the format provided by the authors and unedited

Supplementary Material

1 Results on simplified settings

To build an intuition of why synthetic data is beneficial, we experiment with some toy setups. We assume that to minimise the loss in Equation 1, it is sufficient to minimize the KL divergence between the fair distribution p_f and the combined distribution: p'. We consider the simplified case where the training set is sampled from the fair distribution p_f. The distribution shift arises from the fact that $D_{\texttt{train}}$ is finite; this may cause the actual distribution of $p_{\texttt{train}}$ and p_f to differ.

$$\min_{\theta} \alpha \mathbb{E}_{(\boldsymbol{x},\boldsymbol{a},y)\sim D_{\texttt{train}}} \left(L(f_\theta(\boldsymbol{x}), \boldsymbol{a}, y)\right) + (1-\alpha)\mathbb{E}_{(\boldsymbol{x},\boldsymbol{a},y)\sim \hat{p}} \left(L(f_\theta(\boldsymbol{x}), \boldsymbol{a}, y)\right) \tag{1}$$

1.1 A Bernoulli distribution over a single variable

We assume there is a single variable y we are trying to model that takes one of two values: $y \in \{0, 1\}$. Because $D_{\texttt{train}}$ is finite and consists of $N = |D_{\texttt{train}}|$ samples, its distribution $p_{\texttt{train}}(y)$ (which we refer to as $t(y)$ for conciseness in the following) may drift from p_f (which we refer to as p in the following). We also have a generative model $\hat{p}(y)$. We assume the marginals of these distributions are fixed, so we can only manipulate α.

We want to explore how we should combine $t(y)$ and $\hat{p}(y)$. We can model this by minimizing the KL divergence between p and $p'(y) = t(y)(1-\alpha) + \hat{p}(y)\alpha$: $KL(p||p')$ where α is bounded by $[0, 1]$. For example, if $p = \hat{p}$, then we should just sample from the generative model irrespective of t. Otherwise, there is a trade-off, and we can find the optimal α analytically or experimentally. We compare the distributions with and without generated data for a specific example in Figure 1a. By combining real and generated data we can perfectly match the p distribution.

Next, we consider the more general setting. We evaluate how using a generative model improves performance as we vary (1) the number of samples N from p when creating $D_{\texttt{train}}$; (2) $\hat{p}(y)$ and (3) $p(y)$. For each choice of values, we resample $t(y)$ 1000 times and experimentally find the optimal α. We plot the optimal α as we vary these values in Figure 1b. As can be seen, using generated data is generally helpful (by the fact that $\alpha > 0$). We can also see various properties. First, as N increases (e.g. there is less distribution shift between $D_{\texttt{train}}$ and p), using generated data becomes less helpful (as α goes to 0). Second, generated data is most useful when its distribution is more similar to the true distribution p, but generated data is helpful even in the face of some distribution shift between p and $\hat{p}$. This is perhaps surprising: even in this very simple setting we can see the value of combining the distribution of the training set with another (that of the generated data) in order to mimic the true underlying distribution.

1.2 A Bernoulli distribution over a labeled and hidden variable

The above case does not take into account that we can resample the dataset and use the conditioning variable of the generative model to make $D_{\texttt{train}}$ and $\hat{p}$ more similar to p. However, in reality while we may be able to resample on a labeled variable, there will be many hidden attributes that are important for fairness and generalization. As we do not have labels for these hidden attributes, we *cannot* resample over them to make the distribution more similar to p.

To explore this in a simplistic setting, we proceed as follows. We assume we have two variables

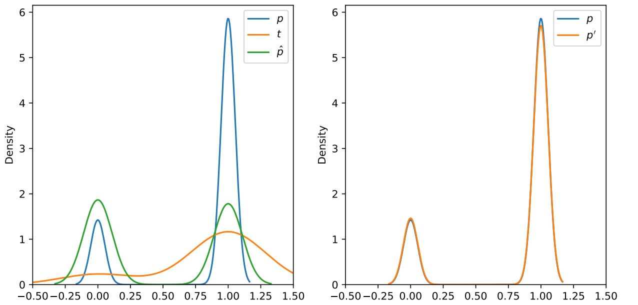

(a) A single run. We set $N = 6, p(y = 0) = 0.2, \hat{p}(y = 0) = 0.5$. To the left we plot the original distributions (real p, generated $\hat{p}$ and the distribution t obtained by sampling 6 points from p). To the right we plot the combined distribution using the optimal α. We can see that in this case, we find $\alpha = 0.1$ is optimal and with the combined distribution, we can perfectly match the true distribution.

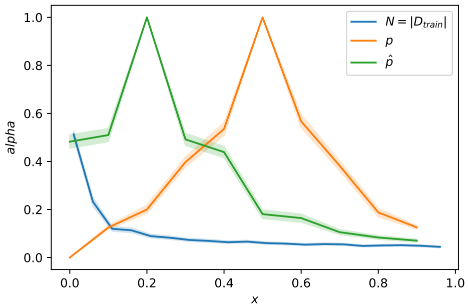

(b) Comprehensive runs. We set $N = 6, p(y = 0) = p = 0.2, \hat{p}(y = 0) = \hat{p} = 0.5$. We then vary each value in turn and plot the optimal α along with the 95% confidence interval. We vary $p, \hat{p}$ between $(0, 1)$ and N between $(0, 100)$. The x-axis plots the normalized value of each variable between (0, 1).

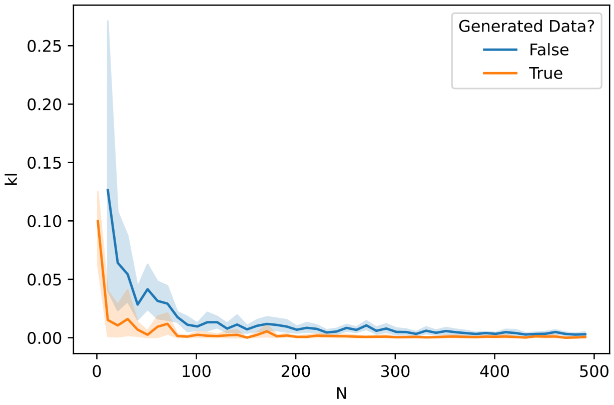

(c) A Bernoulli distribution of two variables.

Figure 1: A Bernoulli distribution. We visualise results for a single run (a) and a comprehensive set of runs (b) to demonstrate that we can leverage generated data to better model the true distribution if there is a distribution shift between the data and true distribution. (c) We vary the number of samples N and experimentally find the optimal $\bar{p}, \bar{t}$. We then compare the KL divergence when using and not using the generated data. We repeat each experiment 10 times for each value of N to obtain the 95% confidence intervals and mean values.

a, y where y is the label and a some hidden attribute which we want to be fair over (and generalise over). Again, they follow a Bernoulli distribution: $a \in \{0, 1\}$ and $y \in \{0, 1\}$.

We condition on the label and can sample arbitrarily according to the label, but this gives a *fixed* distribution over attributes conditioned on the label. More specifically, we can sample arbitrarily to create $\bar{t}(y)$ and $\bar{p}(y)$ (provided $t(y), \hat{p}(y) > 0$) but $\bar{t}(a|y) = t(a|y)$ and $\bar{p}(a|y) = \hat{p}(a|y)$ is fixed. Again, the question is how to optimally combine $t(y, a)$ and $\hat{p}(y, a)$. So now

$$p'(y, a) = t(a|y)\bar{t}(y) + \hat{p}(a|y)\bar{p}(y) \tag{2}$$

$$\sum_y \bar{t}(y) + \bar{p}(y) = 1 \tag{3}$$

We want to minimize the KL divergence between these two distributions, which can be factorized into the sum of the KL divergence between the marginal distribution over a and conditional distribution over y.

$$KL(p'(a, y)||p(a, y)) = KL(p'(y)||p(y)) + KL(p'(a|y)||p(a|y)) \tag{4}$$

This is hard to solve analytically but we can find the optimal distributions of $\bar{t}, \bar{q}$ for a given set of distributions experimentally.

We randomly choose $p = \begin{pmatrix} 0.35 & 0.4 \\ 0.1 & 0.15 \end{pmatrix}$, $p' = \begin{pmatrix} 0.7 & 0.2 \\ 0.05 & 0.05 \end{pmatrix}$ and plot the KL with and without generated data for varying N in Figure 1c. For small values of N, the data distribution is not able to capture the full distribution of p, giving a KL divergence of ∞. However, even for large values of N, using the additional generated data improves the KL divergence. Again, this toy example demonstrates that there is value in leveraging generated data in a low data setting *even* when the training set is sampled from the same distribution as the fair distribution used for evaluation.

1.3 A high dimensional Gaussian Mixture Model (GMM)

Here we demonstrate that additional unlabeled data can improve classification performance in a simple setting. We assume that points come from an underlying GMM where each mixture in the GMM corresponds to a different class. This GMM can have a varying number of dimensions (the number of features) and components (the number of classes). We demonstrate that using a generative model fit to some number of unlabeled points in combination with the labeled points can lead to improved performance of a trained classifier.

Given some number of labeled and unlabeled points, the aim is to train a classifier that performs well on a held out validation set. We do this by fitting a GMM to the unlabeled points and then using the labeled points to determine what class each mode belongs to by minimizing the classification error on the labeled points. We then combine the labeled points and some number of generated points by sampling from the GMM to form the training set. This is used to train the classifier, which is evaluated on the validation set.

We plot the evolution of the GMM being fit to the unlabeled points as the number of unlabeled points varies next to the true, underlying GMM in Figure 2a. We plot the fitted GMM for varying numbers of unlabeled points. With only 500 unlabeled points, the fitted GMM closely matches the true distribution.

We then run the full pipeline including the downstream classifier for varying numbers of labeled (sampled points), generated points, components, and dimensions. We always use 10K unlabeled

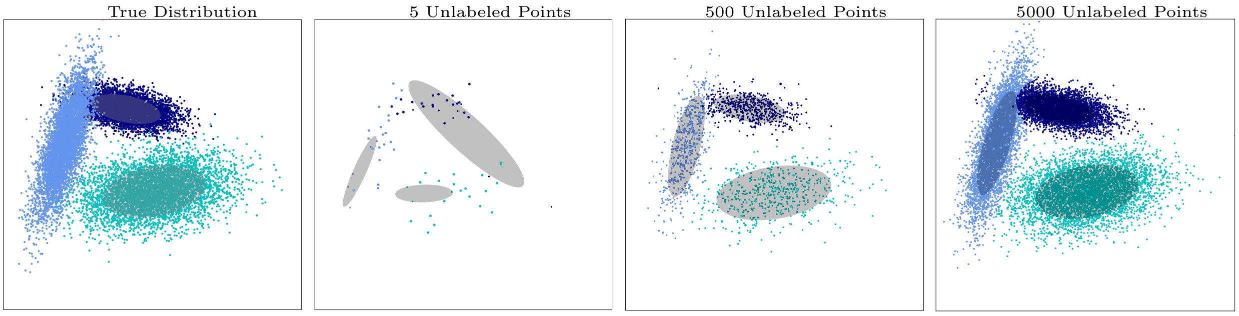

(a) Visualising the fit GMM model for varying numbers of unlabeled points. Large points are labeled points, small points are unlabeled ones. Given only 500 unlabeled points, the fit distribution is similar to the true and with 5000 points, it matches the original.

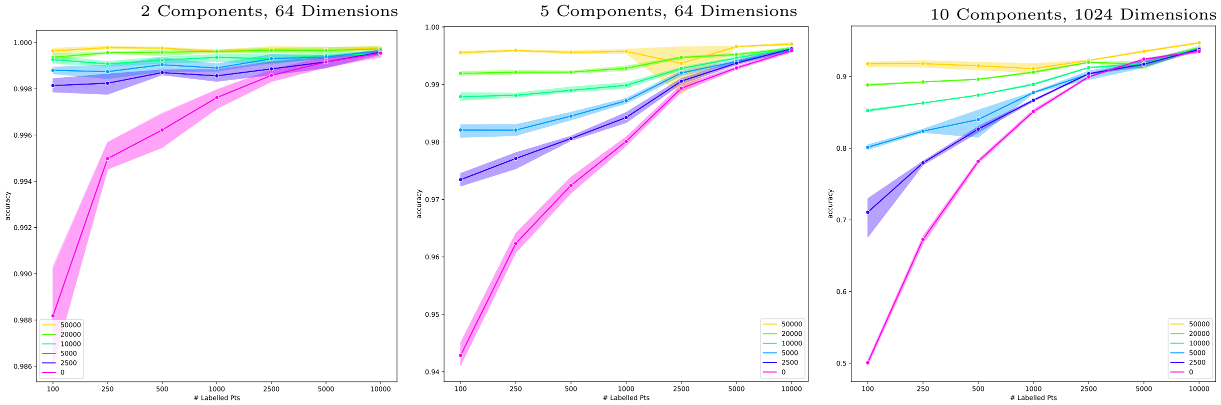

(b) Results for a downstream classifier when using a varying number of generated points from the generative model (which are shown in different colors) and labeled points, which varies along the x-axis. We can see that using additional generated points helps in all these settings (which vary in terms of the number of components and dimensions).

Figure 2: Experiment results with synthetic data from a high-dimensional Gaussian Mixture Model (GMM).

points. To create the GMM, we randomly sample means from the range $[0, 1]$ for each dimension and we create a full covariance metric under a normal distribution with a scale of 0.1. We plot the results in Figure 2b. In these three cases, we can see that using additional generated data improves performance. However, this approach provides limited benefits if (1) the classifier already performs near perfectly using the labeled data; or (2) the mixtures overlap so much that fitting the generative model gives a poor estimate of the underlying distribution.

2 Principal component analysis for spurious correlations

In order to further compare the effect of different augmentation schemes on the features learned by the downstream classifier and investigate why learned augmentations promote better OOD generalization, we design an experiment to account for the simplicity bias [1] within features learned with each augmentation strategy. This phenomenon is linked to the underlying mechanisms that lead models models to rely on spurious correlations to make predictions [1]. In practice, we project N randomly sampled instances from each dataset to the feature space learned by each model and apply the Principal Component Analysis algorithm [2]. We then adopt the number of principal components required to represent different fractions of the variance across all instances projected to the feature spaces as a proxy measure for simplicity in the decision space learned by a model. These

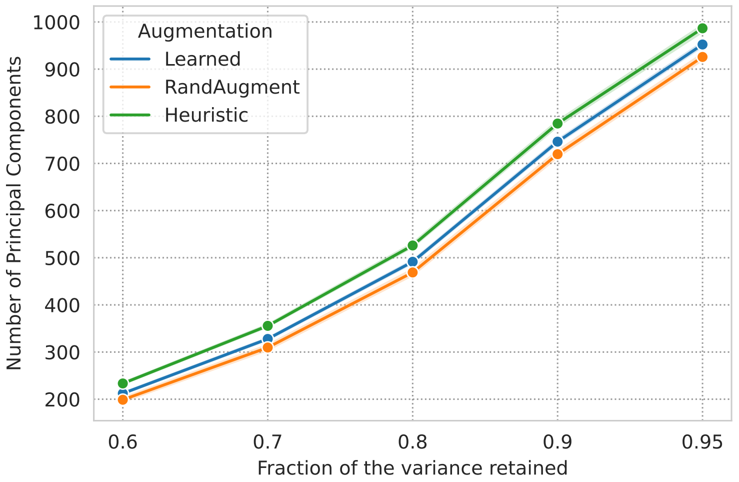

Figure 3: Number of principal components (PCs) required to explain X fraction of the variance after projecting real samples onto the latent space of models trained with heuristic vs. learned and RandAugment augmentations.

feature spaces are induced by models obtained with heuristic and learned augmentations. In Figure 3, we show the average number of components required to retain $\{0.60, 0.70, 0.80, 0.90, 0.95\}$ of the variance of the projected data obtained across five different model initializations for RandAugment, learned, and heuristic augmentations. We observe that for a fixed dataset, features from models trained with learned augmentations and RandAugment require fewer principal components to retain the same fraction of variance for all considered values, indicating that compressing features via removing domain specific cues (*c.f.* Methods, Table G1) yields improved performance. Notably, our findings suggest that learned augmentations enforce models to capture features that strike a better balance between simplicity and predictive power out-of-distribution, i.e. they are simultaneously not overly complex, therefore generalizing well in-distribution, and not too simplistic as in the case of RandAugment, which yield models that generalize *too well* in the vicinity of the training distribution via relying on spurious correlations.

3 Discussion on sampling schemes

In this section, we further discuss and validate the choice of sampling function. First, we motivate different sampling functions within the vanilla loss function (Equation 1) and then evaluate their performance on the downstream classifier to validate the setting used in our main experiments. In particular, we consider variations that aim to reduce the discrepancy between the training data distribution $p_{\texttt{train}}$ and the target "fair" distribution p_f by weighting the losses of training examples. Specifically, the extended loss function is given by

$$R_\alpha(\theta) := \alpha \mathbb{E}_{(\boldsymbol{x},\boldsymbol{a},y)\sim p_{\texttt{train}}} \left[w_1(y,\boldsymbol{a}) \cdot L\left(f_\theta(\boldsymbol{x}), \boldsymbol{a}, y\right)\right] + (1-\alpha)\mathbb{E}_{(\boldsymbol{x},\boldsymbol{a},y)\sim \hat{p}} \left[w_2(y,\boldsymbol{a}) \cdot L\left(f_\theta(\boldsymbol{x}), \boldsymbol{a}, y\right)\right] \quad (5)$$

where the weighting functions w_1 and w_2 adjust the contributions of the real and synthetic examples, respectively. As previously defined, $p_{\texttt{train}}(\boldsymbol{x}, \boldsymbol{a}, y)$ denotes the training distribution over the image, class and attributes, $\hat{p}(\boldsymbol{x}, \boldsymbol{a}, y)$ denotes the approximation of the target fair distribution $p_f(\boldsymbol{x}, \boldsymbol{a}, y)$ based on the generative model, and the steering probability $\alpha \in [0, 1]$ determines the proportion of real data. Let us unpack the loss function term by term.

First, the weighting function $w_1(y, \boldsymbol{a})$ reweighs the losses of the real training examples by the ratio of distributions over sensitive attributes conditioned on the skin condition between the training and the target fair distribution:

$$w_1(y, \boldsymbol{a}) := \left(\frac{p_f(\boldsymbol{a}|y)}{p_{\texttt{train}}(\boldsymbol{a}|y)}\right)^l \tag{6}$$

where $l \geq 0$ ("equality level") controls the degree of penalty. When $l = 1.0$, the first term in Equation 5 equates to the importance weighting [3, 4] with respect to the target fair distribution p_f. Intuitively, because we desire the sensitive attributes to be uniformly distributed per condition in the fair distribution i.e., $p_f(\boldsymbol{a}|y)$ is constant, this weighting function w_1 essentially upweights the losses for examples from under-represented subgroups in the training data (i.e., small $p_{\texttt{train}}(\boldsymbol{a}|y)$), thereby attenuating the discrepancy between $p_{\texttt{train}}$ and p_f. On the other hand, when $l = 0.0$, we recover the default unweighted loss. Introducing this extra hyper-parameter allows us to interpolate between the default loss function and its importance weighted version by choosing $0.0 < l < 1.0$ and also "extrapolate" (i.e., over-penalise the underrepresented groups) by choosing $l > 1.0$.

Second, we introduce the function $w_2(y, \boldsymbol{a})$ that acts as a filtering mechanism for selecting which generated samples to include in the training loss. Our dermatology dataset is dominated by the most prevalent 4 conditions as illustrated in Methods, Figure A2, and we suspect that the benefits of the synthetic data are most pronounced for the remaining less frequent skin conditions. To test this hypothesis, we define $w_2(y, \boldsymbol{a})$ as the indicator function that yields 0.0 if the generated samples belong to the dominant 4 conditions, and otherwise outputs 1.0. We now investigate the effects of the weighting functions w_1 and w_2 on the overall and fairness performance of the downstream classifiers. To generate synthetic data, as in Methods, Section G.3.1, we use the same diffusion model trained on the dermatology dataset at 64×64 resolution for all settings. We use a lower resolution than in the main results for faster iteration, but we expect results to hold at higher resolution too.

Figure 4 shows that, in the absence of any filtering mechanism w_2 (the orange lines), the inclusion of importance weights (equality level = 0.5, 1.0) consistently increases the overall top-3 accuracy and reduces the fairness gap with respect to the sex attribute in both in-distribution and out-of-distribution test datasets. While the improvement in fairness scores through penalisation of under-represented subgroups is expected, the concurrent gains in the overall accuracy comes as a surprise. One possible explanation is that sex and some skin conditions are spuriously correlated, and thus reducing this correlation through importance weighting helps the model learn more generalisable features. However, when combined with the filtering function w_2 (the blue lines in Figure 4), the effects of the importance weighting are more complicated. As before, the fairness gap narrows in most cases. On the other hand, the overall top-3 accuracy only improves in the in-distribution setting but worsens in the out-of-distribution setting. This suggests a subtle interaction between the importance weighting and the filtering.

Finally, we find that the filtering function w_2 (the blue lines) increases the overall top-3 accuracy and lessens the fairness gap in the majority of settings. This result shows that the benefits of the synthetic data in terms of both overall performance and fairness are largely concentrated on the under-represented classes. Additionally, the improvements due to w_2 alone are generally considerably larger than those due to the importance weighting term w_1 on its own. Notably, the best top-3 OOD accuracy is attained with the filtering function alone.

Based on the above analysis, in our experiments, we decided to use the loss function with the filtering function w_2 on the synthetic data but without the importance weighting term w_1 on the real ones for the dermatology datasets. This is because the filtering function w_2 alone confers

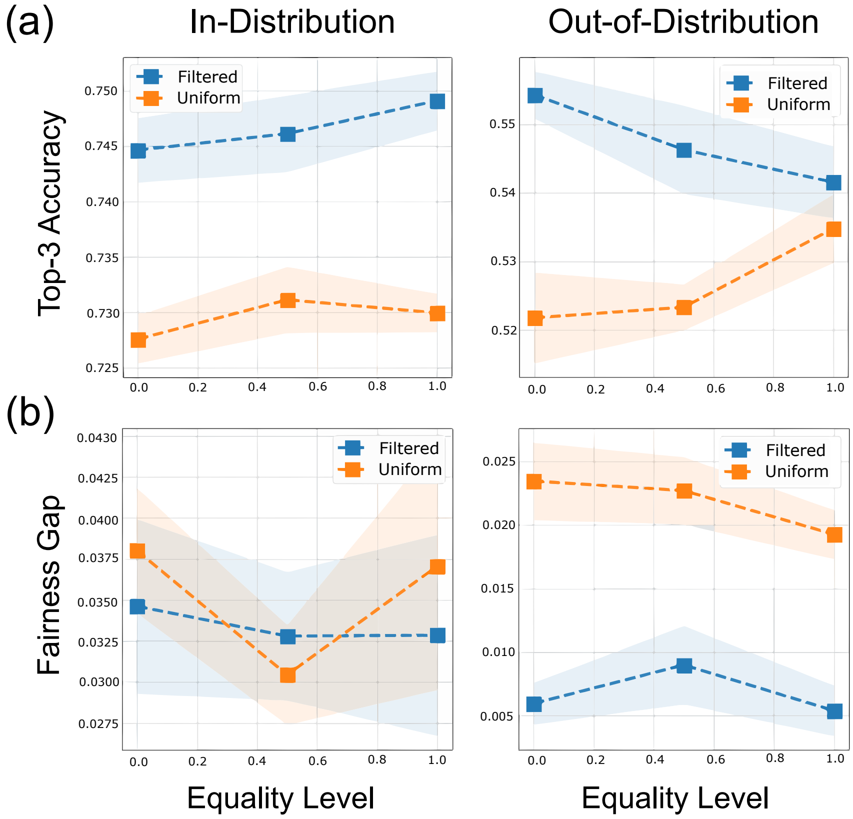

Figure 4: Comparison of different training schemes on our dermatology dataset in terms of (a) the overall top-3 accuracy and (b) the fairness gap with respect to sex. The fairness gap here is measured as the difference in top-3 accuracy between the male and the female subgroups. In particular, we measure the effects of the weighting terms w_1 and w_2 in both in-distribution and out-of-distribution settings. To measure the effects of the weighting function w_1 for the real data, we vary its strength by changing the equality level, l that is on the x-axis (note that when it's zero, it corresponds to no w_1 term, which is the default setting). Secondly, we compare the scenarios with ("*filtered*") and without ("*uniform*") the filtering function w_2 for the synthetic data. The mean and standard deviations of the metrics are calculated over five runs with different random seeds.

considerable improvements in both overall and fairness scores. Although the fairness score can be marginally improved by adding the importance weighting w_1, this comes at the expense of the overall OOD accuracy, which is an important metric in our applications. For the other datasets, as they are more balanced over the label distribution (and histopathology is balanced), we do not use either term.

4 Additional results for dermatology

In this section we provide the results for top-3 accuracy in Figure 5, top-3 balanced accuracy in Figure 6, central best estimate for fairness in Figure 7 and high-risk sensitivity in Figure 8.

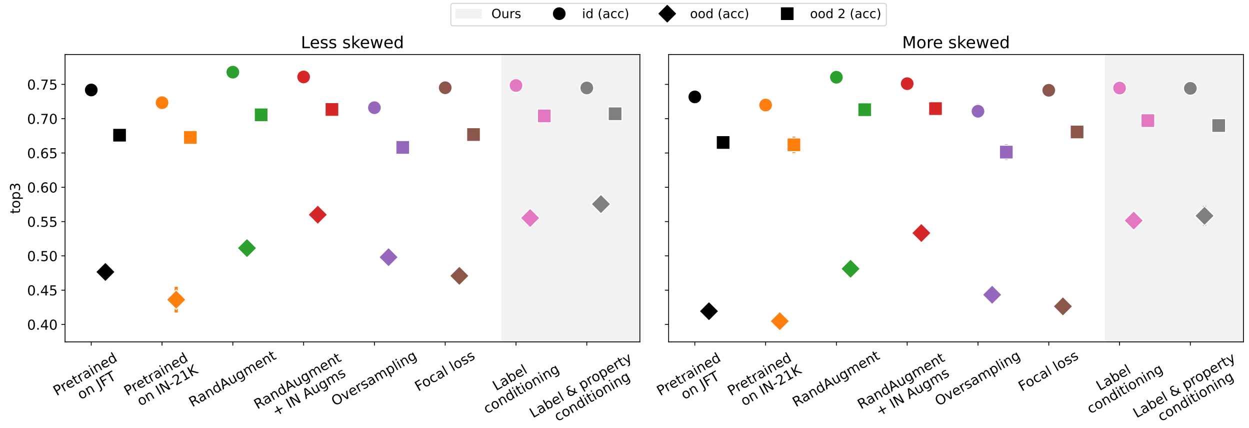

(a) Top-3 accuracy - Sex

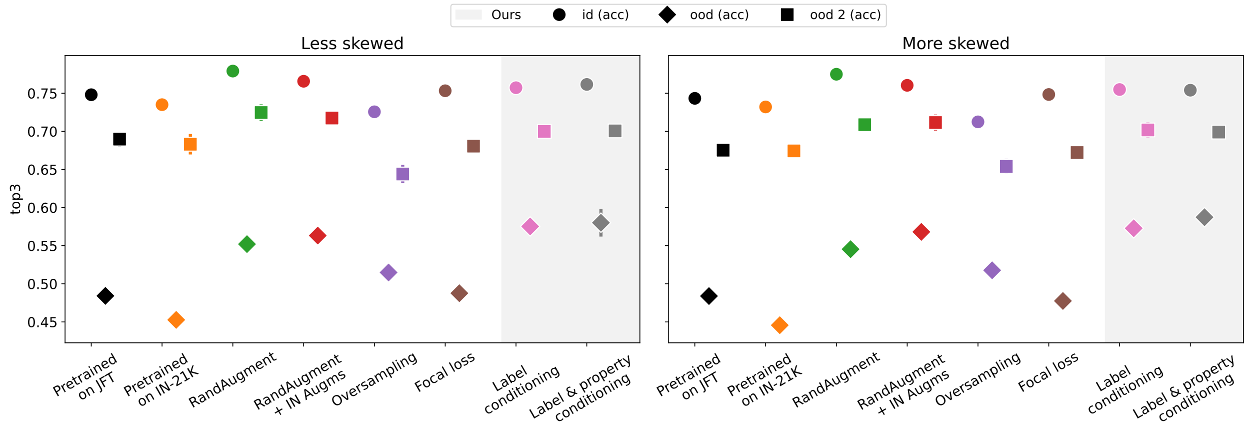

(b) Top-3 accuracy - Skintone

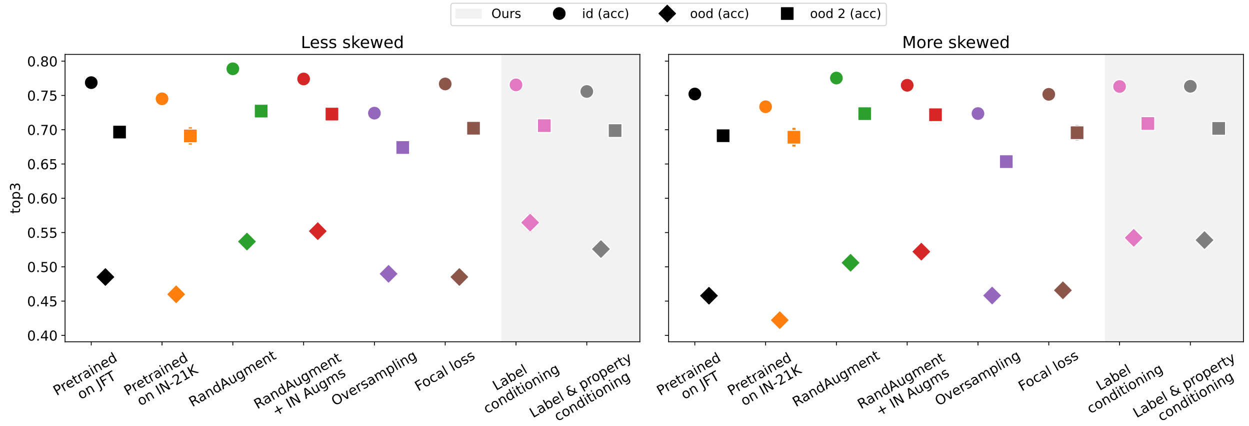

(c) Top-3 accuracy - Age

Figure 5: Top-3 accuracy for dermatology. Higher is better. $n = 1,349$ for id, $n = 6,639$ for ood and $n = 642$ for ood 2. Data are presented as mean values $\pm$ SD across 5 technical replicates.

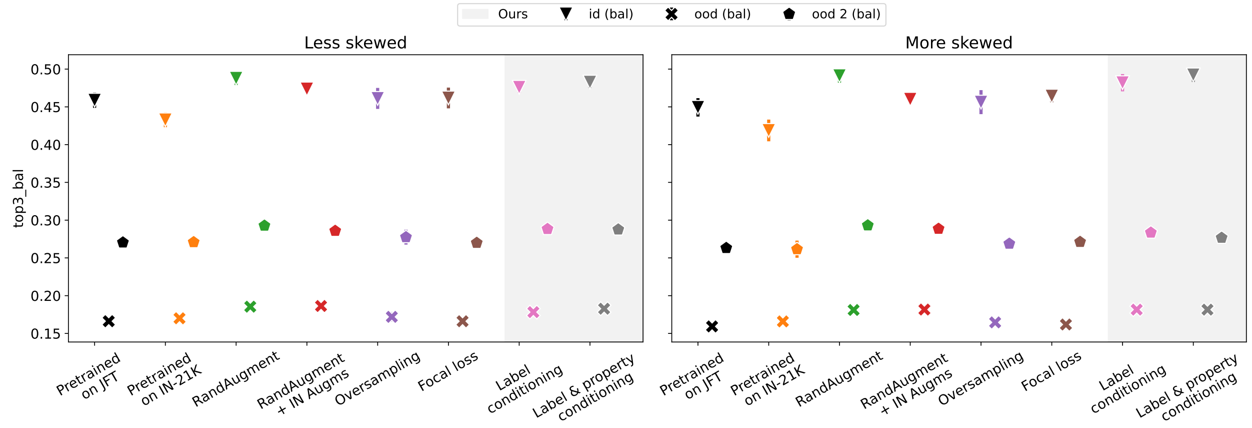

(a) Balanced top-3 accuracy - Sex

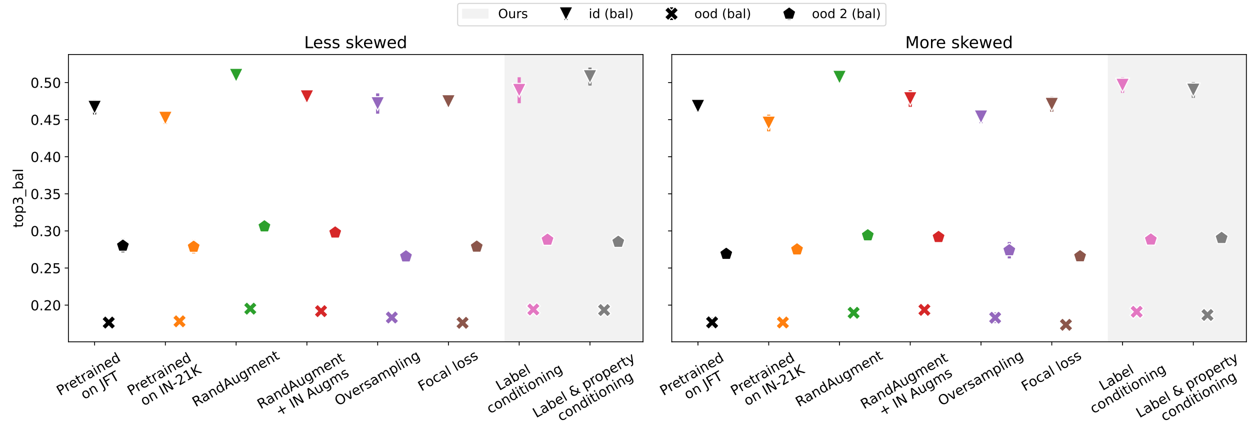

(b) Balanced top-3 accuracy - Skintone

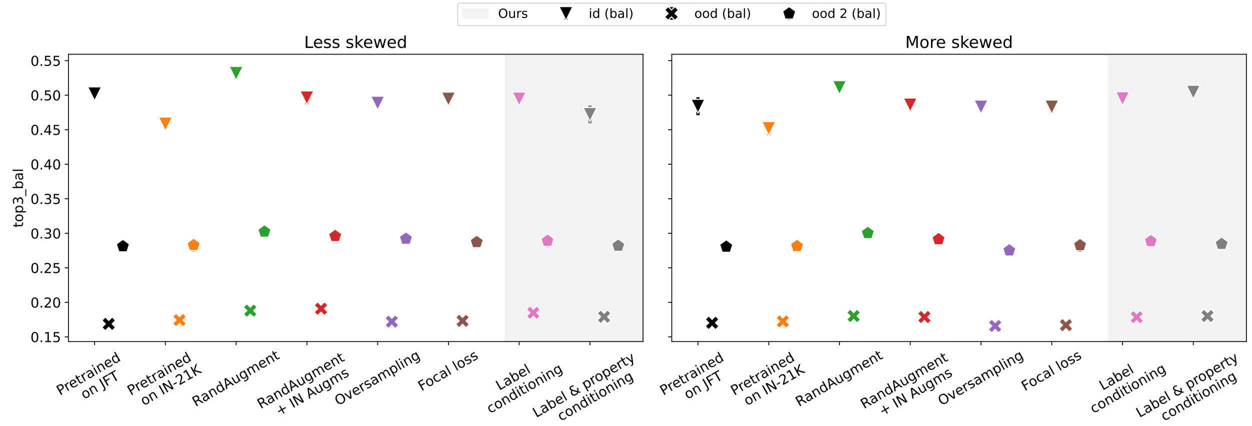

(c) Balanced top-3 accuracy - Age

Figure 6: Top-3 balanced accuracy for dermatology. Higher is better. $n = 1,349$ for id, $n = 6,639$ for ood and $n = 642$ for ood 2. Data are presented as mean values $\pm$ SD across 5 technical replicates.

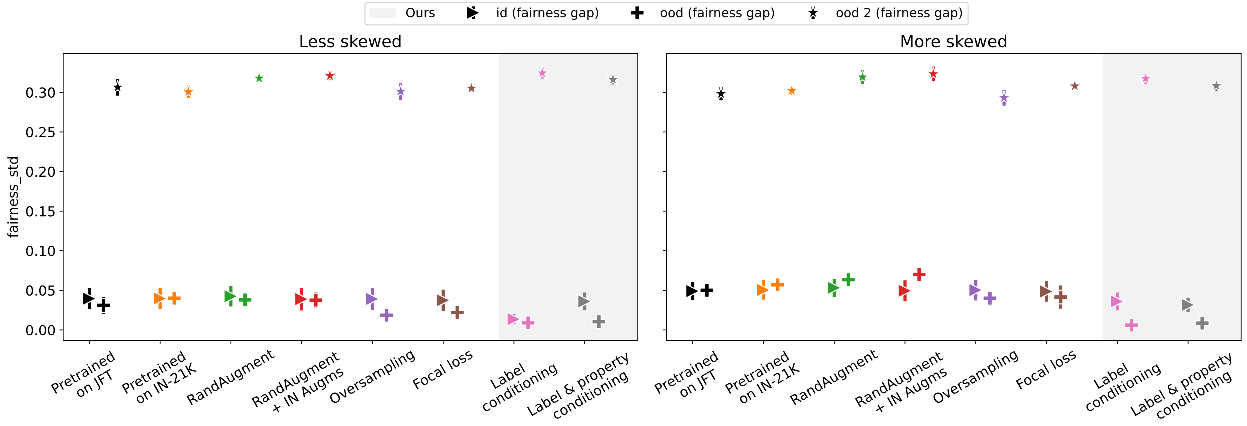

(a) Central best estimate for fairness - Sex

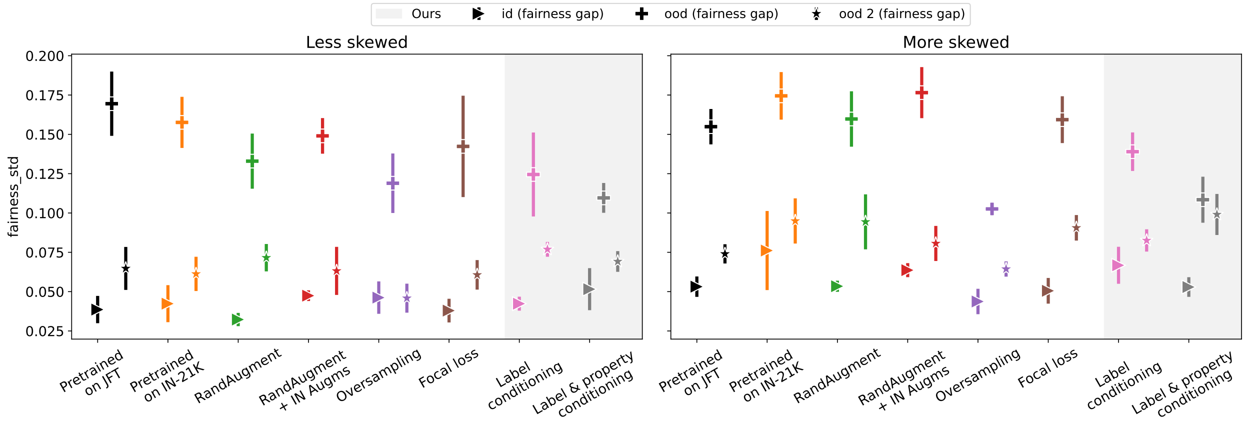

(b) Central best estimate for fairness - Age

Figure 7: Central best estimate for fairness in dermatology. Lower is better. $n = 1,349$ for id, $n = 6,639$ for ood and $n = 642$ for ood 2. Data are presented as mean values $\pm$ SD across 5 technical replicates.

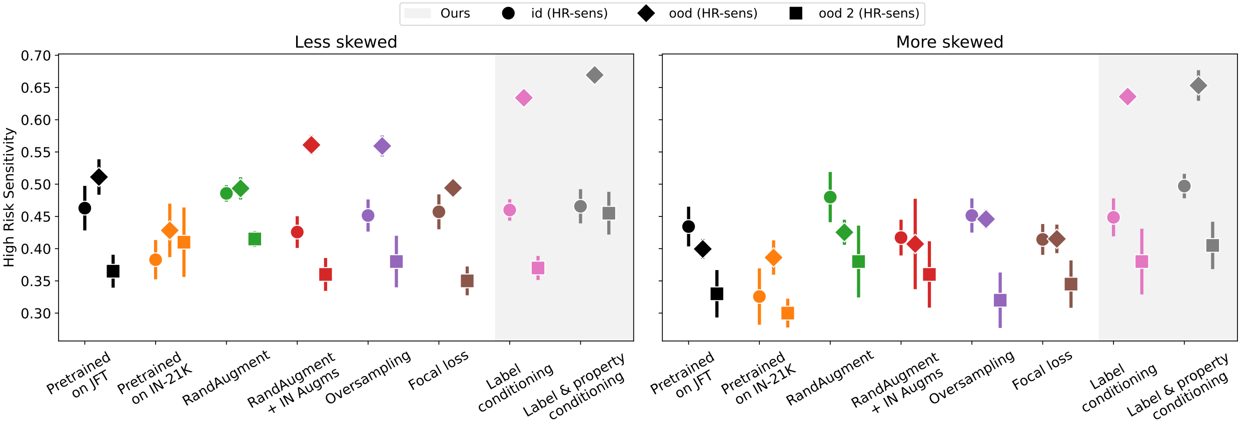

(a) High risk sensitivity - Sex

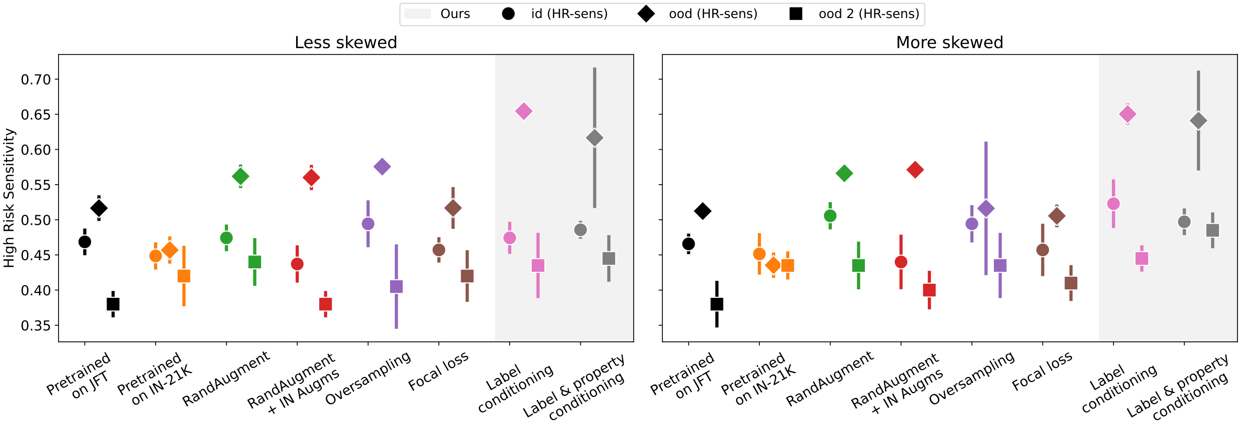

(b) High risk sensitivity - Skintone

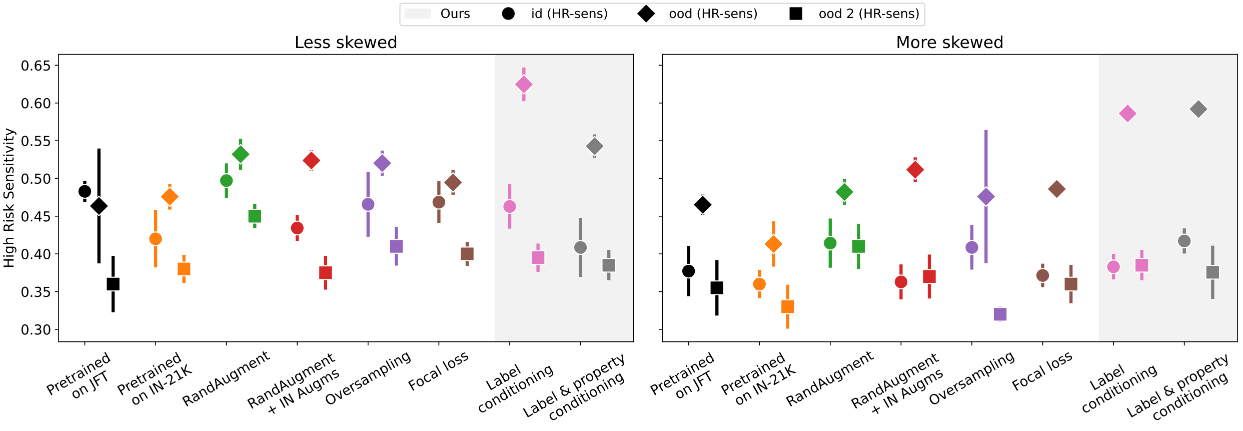

(c) High risk sensitivity - Age

Figure 8: High risk sensitivity for dermatology. Higher is better. $n = 1,349$ for id, $n = 6,639$ for ood and $n = 642$ for ood 2. Data are presented as mean values $\pm$ SD across 5 technical replicates.

References

[1] Harshay Shah, Kaustav Tamuly, Aditi Raghunathan, Prateek Jain, and Praneeth Netrapalli. The pitfalls of simplicity bias in neural networks. *Advances in Neural Information Processing Systems*, 33:9573–9585, 2020.

[2] Hervé Abdi and Lynne J Williams. Principal component analysis. *Wiley interdisciplinary reviews: computational statistics*, 2(4):433–459, 2010.

[3] Daniel G Horvitz and Donovan J Thompson. A generalization of sampling without replacement from a finite universe. *Journal of the American statistical Association*, 47(260):663–685, 1952.

[4] Hidetoshi Shimodaira. Improving predictive inference under covariate shift by weighting the log-likelihood function. *Journal of statistical planning and inference*, 90(2):227–244, 2000.
