# Supplementary material for: Generative models improve fairness of medical classifiers under distribution shifts
Source: Nat Med. 2024 Apr 10;30(4):1166–73. doi: 10.1038/s41591-024-02838-6 (PMC11031395; doi:10.1038/s41591-024-02838-6)
Supplement: Supplementary file 2 — Reporting Summary [file 41591_2024_2838_MOESM2_ESM.pdf]

Corresponding author(s): Ira Ktena
Last updated by author(s): 15/1/2024

Reporting Summary

Nature Portfolio wishes to improve the reproducibility of the work that we publish. This form provides structure for consistency and transparency in reporting. For further information on Nature Portfolio policies, see our Editorial Policies and the Editorial Policy Checklist.

Statistics

For all statistical analyses, confirm that the following items are present in the figure legend, table legend, main text, or Methods section.

n/a	Confirmed	
☐	☒	The exact sample size (*n*) for each experimental group/condition, given as a discrete number and unit of measurement
☐	☒	A statement on whether measurements were taken from distinct samples or whether the same sample was measured repeatedly
☐	☒	The statistical test(s) used AND whether they are one- or two-sided<br>*Only common tests should be described solely by name; describe more complex techniques in the Methods section.*
☐	☒	A description of all covariates tested
☒	☐	A description of any assumptions or corrections, such as tests of normality and adjustment for multiple comparisons
☐	☒	A full description of the statistical parameters including central tendency (e.g. means) or other basic estimates (e.g. regression coefficient) AND variation (e.g. standard deviation) or associated estimates of uncertainty (e.g. confidence intervals)
☐	☒	For null hypothesis testing, the test statistic (e.g. *F*, *t*, *r*) with confidence intervals, effect sizes, degrees of freedom and *P* value noted<br>*Give P values as exact values whenever suitable.*
☒	☐	For Bayesian analysis, information on the choice of priors and Markov chain Monte Carlo settings
☒	☐	For hierarchical and complex designs, identification of the appropriate level for tests and full reporting of outcomes
☒	☐	Estimates of effect sizes (e.g. Cohen's *d*, Pearson's *r*), indicating how they were calculated

Our web collection on statistics for biologists contains articles on many of the points above.

Software and code

Policy information about availability of computer code

Data collection	No software was used for data collection.
Data analysis	Several major components of our work are available in open source repositories such as the \href{https://github.com/deepmind/dm-haiku/blob/main/haiku/_src/nets/resnet.py}{Haiku} library. The codebase and pretrained weights for BiT models are available at \href{https://github.com/google-research/big_transfer}{Big Transfer GitHub}. The guided diffusion implementation is based on the \href{https://github.com/hojonathanho/diffusion/blob/1e0dceb3b3495bbe19116a5e1b3596cd0706c543/diffusion_tf/diffusion_utils_2.py}{Diffusion Github} repository. All<br>experiments and implementation details are described in sufficient detail in the Methods to support replication with non-proprietary libraries. We further provide model weights for generative models trained on the public CAMELYON and Fitzpatrick 17k datasets in \url{https://github.com/google-deepmind/augmentations_medical_images}, along with inference code to reproduce the final results using these models.

For manuscripts utilizing custom algorithms or software that are central to the research but not yet described in published literature, software must be made available to editors and reviewers. We strongly encourage code deposition in a community repository (e.g. GitHub). See the Nature Portfolio guidelines for submitting code & software for further information.

Data

Policy information about availability of data

All manuscripts must include a data availability statement. This statement should provide the following information, where applicable:

- Accession codes, unique identifiers, or web links for publicly available datasets
- A description of any restrictions on data availability
- For clinical datasets or third party data, please ensure that the statement adheres to our policy

The de-identified teledermatology data used in this study are not publicly available due to restrictions in the data-sharing agreement. The data is available for non-commercial purposes for an administrative fee, providing that the requesting entity can comply with applicable laws and the privacy policy of the data provider. Please contact \url{dermatology-research@google.com} who can help forward any requests to the source, with a response timeframe of maximum two weeks. We further performed experiments on the public \href{https://github.com/mattgroh/fitzpatrick17k}{Fitzpatrick 17k} dermatology dataset. Data used in training and evaluation of chest radiology classification, including \href{https://stanfordmlgroup.github.io/competitions/chexpert/}{CheXpert}, and \href{https://www.kaggle.com/nih-chest-xrays/data}{ChestX-ray14} are publicly available. Data used for in-distribution fine-tuning and evaluation of pathology metastases detection is publicly available on the \href{https://camelyon16.grand-challenge.org/Data/}{CAMELYON} challenge website. Moreover, ImageNet-21K~\cite{russakovsky2015imagenet} and JFT-300M~\cite{Sun17} have been used for pretraining of baseline supervised models. ImageNet-21K is publicly available at ImageNet website, but the JFT-300M dataset is not publicly available due to restrictions in the data-sharing agreement.

Research involving human participants, their data, or biological material

Policy information about studies with human participants or human data. See also policy information about sex, gender (identity/presentation), and sexual orientation and race, ethnicity and racism.

Reporting on sex and gender	For Chest X-ray and dermatology datasets, self-reported sex has been used for the fairness analysis. We share aggregated sex information in the "Methods". For histopathology data, sex/gender information is not available.
Reporting on race, ethnicity, or other socially relevant groupings	For Chest X-ray, we use the primary race labels obtained from \url{https://stanfordaimi.azurewebsites.net/datasets/192ada7c-4d43-466e-b8bb-b81992bb80cf} for the in-distribution CheXpert dataset.
Population characteristics	Our analysis was performed on dermatology data from individuals aged 15-90 years old, while the chest radiology analysis was performed on individuals 18-90 years old. We provide the age distribution of participants in the "Methods" section.
Recruitment	We did not recruit individuals for this study; all datasets used were previously collected and have been used by prior studies.
Ethics oversight	Given that no new data was collected for the purposes of this study, there was no approval of the study protocol required.

Note that full information on the approval of the study protocol must also be provided in the manuscript.

Field-specific reporting

Please select the one below that is the best fit for your research. If you are not sure, read the appropriate sections before making your selection.

☒ Life sciences ☐ Behavioural & social sciences ☐ Ecological, evolutionary & environmental sciences

For a reference copy of the document with all sections, see nature.com/documents/nr-reporting-summary-flat.pdf

Life sciences study design

All studies must disclose on these points even when the disclosure is negative.

Sample size	Test sets for all 3 medical tasks have been previously published and the sample size has therefore been shown to be sufficient for estimation of model diagnostic accuracy with acceptable uncertainty. Sample sizes were defined based on (i) Koh et al., ICML (2021) for histopathology (ii) Azizi et al., Nat Biomed Eng. (2023) for chest-radiology and dermatology datasets. The evaluation sample sizes were all >1,000 and chosen so that conclusions can be reliably reached.
Data exclusions	Datasets used reflect those used in previous papers, no additional data exclusions were applied except in the case of dermatology where training datasets were skewed to stress test generalization capabilities of the model. However, the exact process is explicitly described in the manuscript.
Replication	We are making code available to ensure that the work can be replicated externally, internal tests for replication are a standard engineering practice at Google. Across all experiments we performed 5 replicates, in order to ensure that we capture variability in the model initialization as is standard practice in prior ML studies on these datasets (see Koh et al., ICML 2021).
Randomization	Randomization was performed to create the train, validation and test splits of the different datasets used in the study.
Blinding	N/A

Reporting for specific materials, systems and methods

We require information from authors about some types of materials, experimental systems and methods used in many studies. Here, indicate whether each material, system or method listed is relevant to your study. If you are not sure if a list item applies to your research, read the appropriate section before selecting a response.

Materials & experimental systems

n/a	Involved in the study
☒	☐ Antibodies
☒	☐ Eukaryotic cell lines
☒	☐ Palaeontology and archaeology
☒	☐ Animals and other organisms
☒	☐ Clinical data
☒	☐ Dual use research of concern
☒	☐ Plants

Methods

n/a	Involved in the study
☒	☐ ChIP-seq
☒	☐ Flow cytometry
☒	☐ MRI-based neuroimaging
